# Neural decoding with visual attention using sequential Monte Carlo for leaky integrate-and-fire neurons

**DOI:** 10.1371/journal.pone.0216322

**Published:** 2019-05-14

**Authors:** Kang Li, Susanne Ditlevsen

**Affiliations:** 1 Department of Psychology, University of Copenhagen, Copenhagen, Denmark; 2 Department of Mathematical Sciences, University of Copenhagen, Copenhagen, Denmark; SUNY Downstate MC, UNITED STATES

## Abstract

How the brain makes sense of a complicated environment is an important question, and a first step is to be able to reconstruct the stimulus that give rise to an observed brain response. Neural coding relates neurobiological observations to external stimuli using computational methods. Encoding refers to how a stimulus affects the neuronal output, and entails constructing a neural model and parameter estimation. Decoding refers to reconstruction of the stimulus that led to a given neuronal output. Existing decoding methods rarely explain neuronal responses to complicated stimuli in a principled way. Here we perform neural decoding for a mixture of multiple stimuli using the leaky integrate-and-fire model describing neural spike trains, under the visual attention hypothesis of probability mixing in which the neuron only attends to a single stimulus at any given time. We assume either a parallel or serial processing visual search mechanism when decoding multiple simultaneous neurons. We consider one or multiple stochastic stimuli following Ornstein-Uhlenbeck processes, and dynamic neuronal attention that switches following discrete Markov processes. To decode stimuli in such situations, we develop various sequential Monte Carlo particle methods in different settings. The likelihood of the observed spike trains is obtained through the first-passage time probabilities obtained by solving the Fokker-Planck equations. We show that the stochastic stimuli can be successfully decoded by sequential Monte Carlo, and different particle methods perform differently considering the number of observed spike trains, the number of stimuli, model complexity, etc. The proposed novel decoding methods, which analyze the neural data through psychological visual attention theories, provide new perspectives to understand the brain.

## Introduction

Neural coding is the science of characterizing the relationship between a stimulus presented to a neuron or an ensemble of neurons, and the neuronal responses [1]. Neural encoding refers to the map from stimulus to response, i.e., how the neurons respond to a specific stimulus. For example, if we can construct an encoding model, it can be used to predict responses to other stimuli. Neural decoding refers to the reverse map, from response to stimulus, and the challenge is to reconstruct a stimulus, or certain aspects of that stimulus, from the evoked spike train. Neural coding is extensively studied in computational neuroscience.

Our aim is to decode complicated multiple stochastic stimuli from neural spike trains. We combine biophysical spiking neural models with visual attention theories, bridging computational neuroscience and cognitive psychology. Following the visual attention model [2, 3], attention to complicated multiple stimuli is viewed as probability mixtures. The two visual search mechanisms in psychology, the parallel and the serial processing [4], are employed for decoding neuron ensembles.

The goal of this paper is to develop, explore and compare various decoding methods based on sequential Monte Carlo for multiple stimuli in a visual attention setting.

### Neural decoding

Given neurobiological observations, a decoding algorithm aims at reconstructing the unknown stimulus information encoded by the neural system. Neural decoding plays an important role in understanding the mechanisms of neurons and the brain. Well-performing algorithms of decoding constitute necessary components of brain-machine interfaces [5, 6]. Different methods have been explored to study neural decoding. Some methods focus on regression-related approaches building linear models between spike trains and the corresponding stimulus by optimal linear estimation (OLE) [7, 8]. Machine learning methods are also employed to stimulus decoding, such as artificial neural networks [9], kernel regression [10], and a recently developed approach using kernel-based neural metrics [11]. These methods employ general statistical techniques and omit the specific spike-generating mechanism of the neural response. On the other hand, stimulus decoding may directly employ spiking neural models that describe the spike generating mechanisms from stimuli [12–15]. Various encoding models can be used. Approximate methods using point processes treat the spikes in a spike train as sequential random events, which can be equivalently formulated as generalized linear models (GLM) for model fitting [15, 16]. Meanwhile, there are also biophysically motivated methods like integrate-and-fire models, which study the stochastic evolution of the membrane potential. In decoding tasks, these encoding models are used in the posterior distribution to infer the most likely stimuli. Decoding of constant stimulus can be obtained from the posterior distribution using maximum a posteriori (MAP) or Monte Carlo methods. The decoding of temporal stimuli can be discretized as a sequence of constant decoding tasks, which can be solved by Kalman filtering [17] or particle sequential Monte Carlo methods [18–21].

### Modeling visual attention

#### Stimulus mixture and probability mixing

We define a stimulus mixture to be multiple non-overlapping stimuli inside the receptive field of a neuron. We assume that the neuronal response to a stimulus mixture follows the probability-mixing model [2, 22], where the neuron responds at any given time to only one of the single stimuli in the mixture with certain probabilities. In [3] data from MT neurons in macaque monkeys are analyzed, and the probability-mixing model appears to be more in agreement with data compared to the competing response-averaging model. The probability-mixing model enables us to accurately perform decoding, i.e., to recover the single stimulus that caused the response.

#### Neural behaviour during parallel and serial processing

The two opposing visual search mechanisms of parallel and serial processing have been long debated in psychology, and empirical behavioral experiments have shown evidence supporting both mechanisms [4, 23–25]. According to serial processing, multiple objects are processed sequentially by the brain, such that only one object is being processed at any given time point, and according to parallel processing, multiple objects are processed concurrently in parallel. We explain parallel and serial processing from a neural perspective, based on the Neural Theory of Visual Attention (NTVA) [2] stating that a neuron can only represent a single object at any time. It follows that in serial processing, all neurons in the high level visual cortex must respond to the same single object at any given time, whereas in parallel processing, neurons can split the attention, such that some neurons represent one object and others represent other objects at the same time. Here we do not aim to select one mechanism over the other. We will assume either mechanism, and perform decoding in both cases.

#### Stochastic stimulus

A stimulus is stochastic if it contains strong and inevitable noise apart from a deterministic trend, for example a stimulus described by a stochastic diffusion process. Decoding stochastic stimuli requires obtaining parameter estimates as well as recovering the stochastic realization of the stimulus at all time steps. The stimulus may represent the strength of light or sound, the position of objects, etc, and these signals are more realistically described by stochastic processes than deterministic functions. Here we consider mixtures of stochastic stimuli evolving continuously over time following Ornstein-Uhlenbeck processes with unknown parameters.

#### Markov attention switching

Consider the case where a neuron is responding to a mixture of multiple stimuli following the probability-mixing model. One possible situation is that the neuronal response is fixed, responding to the same stimulus component in the mixture during the whole trial. Another more probable situation for long trials is that the neuron switches between stimuli, only responding to a certain stimulus for some time whereafter it switches to another stimulus, and the switching is random following a Markov chain with certain transition probabilities. During the process, the neuron can only respond to one single stimulus in the mixture at a given time, according to probability-mixing and NTVA.

### Leaky integrate-and-fire model

The leaky integrate-and-fire (LIF) models are simple diffusion models for the dynamics of the membrane potential in single neurons [26, 27], the most common being an Ornstein-Uhlenbeck (OU) process with constant conductance, leak potential, and diffusion coefficient. The model can be extended by incorporating post-spike currents with a spike-response kernel function [28]. Here we first focus on a bursting response kernel [29] (rhythmic spiking), then we try two other kernels causing a decay of the spiking rate (adaptation) and a delay of spike formation (refractory period). These kernels have been used to study parameter estimation in LIF models responding to a plurality of stimuli in the same visual attention framework [22]. The likelihood function of an observed spike train was computed using different approaches by numerically solving either the Fokker-Planck partial differential equations (PDE) or the Volterra integral equations. In this study we only focus on the PDE method, which provides the best solution when considering the trade-off between accuracy and computational burden [22].

## Models and methods

### Encoding model: The leaky integrate-and-fire model

The encoding model is a standard LIF model extended with a spike response kernel, and is the same as used in [22]. We will briefly repeat it here for convenience. The evolution of the membrane potential is model by the solution to the stochastic differential equation:

$$\begin{array}{cc} \text{d}X(t) & =b(X(t),t)\text{d}t+\sigma \text{d}W(t)\\ & =(-a(X(t)-\mu )+I(t)+H(t))\text{d}t+\sigma \text{d}W\left(t\right),\\ X(0) & =x_{0}\;;\;X\left(t_{j}^{+}\right)=x_{0}\\ t_{j} & =\inf\left\{t>t_{j-1}\;:\;X\left(t\right)=x_{th}\right\}\;\;\text{for}\;j\geq 1,\;t_{0}=0, \end{array}$$ (1)

where $t_{j}^{+}$ denotes the right limit taken at *t*_*j*_. The drift term *b*(⋅) contains three currents: the leak current −*a*(*X*(*t*) − *μ*), where *a* > 0 is the decay rate and *μ* is the reversal potential, the stimulus driven current *I*(*t*), and the post-spike current *H*(*t*). The potential *X*(*t*) evolves until it reaches the threshold, *x*_*th*_, where it resets to *x*_0_. The membrane potential *X*(*t*) is not measured, only the spike times {*t*_1_, *t*_2_, …} are observed. Thus, the scaling of *X* is arbitrary, and we can use any values for threshold and reset. We set *x*_0_ = 0 and *x*_*th*_ = 1 such that *X* is measured in units of the distance between reset and spike threshold. The noise is modelled by the standard Wiener process, *W*(*t*), with diffusion parameter, *σ* > 0.

The stimulus current *I*(*t*) is shaped from the external stimulus *S*(*t*) through a stimulus kernel *k*_*s*_(*t*); $I\left(t\right)=\int_{-\infty }^{t}k_{s}\left(t-s\right)S\left(s\right)\text{d}s$. The post-spike current arises from past spikes convoluted with a response kernel *k*_*h*_(*t*); $H\left(t\right)=\int_{-\infty }^{t}k_{h}\left(t-s\right)I\left(s\right)\text{d}s$. Here $I\left(s\right)=\sum_{\tau \in \{t_{1},t_{2},\ldots \}}\delta \left(s-\tau \right)$ represents the spike train, where *δ*(⋅) denotes the Dirac delta function.

We assume a stimulus kernel without delay, such that *k*_*s*_(*t*) = *δ*(*t*), implying that *I*(*t*) = *S*(*t*). The response kernel is assumed to be the difference of two exponentials decaying over time,

$$\begin{array}{c} k_{h}\left(t\right)=\eta_{1}e^{-\eta_{2}t}-\eta_{3}e^{-\eta_{4}t} \end{array}$$ (2)

with four positive parameters, *η* = (*η*_1_, *η*_2_, *η*_3_, *η*_4_). By adjusting the parameters, different kernels are obtained. Three types of kernels are used here, described in Table 1 and illustrated later in the Results section. In the center panels example spike trains generated from the different kernels and different stimuli are illustrated.

**Table 1:** Characteristics of response kernels used in the encoding model.

Kernel	Description	Parameter	Interpretation
Bursting	first positive, then negative, then vanishing	*η*_1_ > *η*_3_, *η*_2_ > *η*_4_	recent spikes have excitatory effects, accumulation of spikes has inhibitory effects, resulting in rhythmic spiking with bursts
Decaying	first negative, then vanishing	*η*_1_ = 0, *η*_3_, *η*_4_ small	inhibitory effects are small but long-lasting, making the firing rate decay slowly over time
Delaying	first negative, then positive, then vanishing	*η*_1_ < *η*_3_, *η*_2_ < *η*_4_	recent spikes have inhibitory effects, accumulation of spikes has excitatory effects, preventing short interspike intervals (refractory period)

### Likelihood of an observed spike train

Suppose there are a total of *K* stimuli inside the receptive field of the neuron, denoted by *S* = (*S*^1^, …, *S*^*K*^). Let *Y* = (*Y*^1^, …, *Y*^*M*^) denote *M* spike trains. The realizations of stimuli and spike trains are respectively *s* = (*s*^1^, …, *s*^*K*^) and *y* = (*y*^1^, …, *y*^*M*^). According to the probability-mixing encoding model, the stimulus-driven current, *I*(*t*), follows a probability mixture:

$$\begin{array}{c} I\left(t\right)=S^{k}\left(t\right),\;\text{with}\;\text{probality}\;\alpha_{k}, \end{array}$$ (3)

for *k* = 1, …, *K*, where $\sum_{k=1}^{K}\alpha_{k}=1$. Then the probability of a spike train *y*^*m*^ generated under the exposure of the *K* stimuli is also a mixture distribution,

$$\begin{array}{c} p\left(y^{m}|s\right)=\sum_{k=1}^{K}\alpha_{k}p\left(y^{m}|s^{k}\right), \end{array}$$ (4)

where *p*(*y*^*m*^|*s*^*k*^) is the probability of generating spike train *y*^*m*^ from the single stimulus *s*^*k*^. It equals the product of the probability densities of all spike times within *y*^*m*^ = (*t*_1_, *t*_2_, …), where the dependence between spike times is accounted for by conditioning on the history of past spike times, $H_{t_{i-1}}^{m}$,

$$\begin{array}{c} p\left(y^{m}|s^{k}\right)=\prod_{i}g\left(t_{i}|s^{k},H_{t_{i-1}}^{m}\right), \end{array}$$ (5)

where $g(t|s^{k},H_{t_{i-1}})$ is the conditional probability density of spiking at time *t* given the *k*th stimulus and the spike history up to the previous spike time *t*_*i*−1_. The probability density *g*(⋅) can be obtained from the density of the first-passage time of model (1), which we calculate by numerically solving the Fokker-Planck equation; see Appendix I: Probability of ISIs. If the *M* spike trains *Y* = (*Y*^1^, …, *Y*^*M*^) are assumed independent, then the likelihood for *y* = (*y*^1^, …, *y*^*M*^) is

$$\begin{array}{c} p\left(y|s\right)=\prod_{m=1}^{M}p\left(y^{m}|s\right)=\prod_{m=1}^{M}\sum_{k=1}^{K}\alpha_{k}p\left(y^{m}|s^{k}\right). \end{array}$$ (6)

### Decoding of stochastic stimulus mixtures with Markov switching

We consider stochastic stimulus mixtures with Markov attention switching, described by stochastic processes with unknown parameters. The focus is both on estimating parameters governing the law of the *k*th stimulus, as well as decoding of the stochastic realization of the stimulus. We discretize the time interval of a trial in smaller intervals of length *v*, and assume that the neurons can only switch attention between intervals, but will attend the same stimulus during any of these small intervals. [30] found that sustained attention naturally fluctuates with a periodicity of 4–8 Hz, thus, at most switching attention after 125*ms*. In the simulations presented later, we set *v* = 100*ms*. Denote by *C*_*n*_ the index of the attended stimulus at the *n*th time point, *C*_*n*_ ∈ {1, …, *K*}, *n* = 1, …, *N*, such that *vN* is the length of the total observation interval, and let *S*_*n*_ denote the stochastic realization of the attended stimulus at the *n*th time point. In the decoding algorithm, it is assumed that *S*_*n*_ is constant, thus approximating the stochastic stimulus process by a piecewise constant process. Assume the neuron switches attention between two consecutive time intervals following a Markov chain with transition probability matrix (TPM) **Γ**. Denote the elements of **Γ** by λ_*kl*_ for *k*, *l* = 1, …, *K*. Thus, λ_*kl*_ = *P*(*C*_*n*_ = *l*|*C*_*n*−1_ = *k*) is the probability that at time *n* the attended stimulus is *S*^*l*^, given that the neuron attended stimulus *S*^*k*^ at time *n* − 1.

The stochastic stimuli are described by Ornstein-Uhlenbeck (OU) processes. For a mixture of *K* stimuli *S* = (*S*^1^, …, *S*^*K*^), the *k*th stimulus component is governed by the stochastic differential equation (SDE):

$$\begin{array}{c} \text{d}S^{k}\left(t\right)=\left[\beta^{k}-S^{k}\left(t\right)\right]\text{d}t+\gamma \text{d}W\left(t\right), \end{array}$$ (7)

where *β*^*k*^ and *γ* are parameters, and *W*(*t*) is a standard Wiener process. Only the drift parameter *β*^*k*^ is stimulus specific, the diffusion parameter *γ* is assumed to be the same for all stimuli in the mixture.

The parameters describing the stimulus are unknown, namely *γ*, *β* = (*β*^1^, …, *β*^*K*^) and the TPM **Γ**, so that *θ* = (*γ*, *β*, **Γ**). The parameter space is thus Θ = **R**_+_ × **R**^*K*^ × *Ω*, where *Ω* is the space of *K* × *K* stochastic matrices. For simplicity, the mixture number *K* is assumed to be known. If *K* is unknown, then the algorithm is run with different *k* = 1, 2, …, and the *k* that minimizes the BIC is chosen. We focus on various Monte Carlo techniques for decoding, including the bootstrap filter, the auxiliary particle filter with parameter learning, fixed-lag and fixed-interval smoothing, etc; see [31] for a review of such methods. The goal is to decode the stochastic realization of *S*_*n*_ for *n* = 1, …, *N*. We will present online methods, where parameter estimates are updated sequentially as observations become available. We also explore smoothing techniques, where some delay is allowed before the stimulus is reported.

### Sequential Monte Carlo methods

We will now establish sequential Monte Carlo methods for decoding. In Table 2 below, we summarize the methods that are developed and compared. The details are described in the following sections.

**Table 2:** Summary of methods.

**Single spike trains**		
	Methods	Comments
	BF, APF	Compared with the Bootstrap Filter (BF), the Auxiliary Particle filter (APF) applies two-stage resampling with auxiliary variables and performs parameter learning along time.
**Multiple spike trains**		
	Methods	Comments
Serial	BF, APF	In serial processing, multiple simultaneously recorded spike trains are analyzed.
Parallel	iBF, mBF, iAPF, mAPF	In parallel processing, single spike trains are either decoded independently and results are merged (i-), or all spike trains are decoded together and the marginal likelihood is computed (m-).
**Extensions**		
	Methods	Comments
APF resampling	APFg	Resample using geometric mean of likelihood.
Smoothing	-F	Online filtering.
	-lag	Fixed-lag smoothing by marginalization.
	-FB	Fixed-interval smoothing using Forward-Backward algorithm.
Continuous-time switching	-	Discretization methods can approximate continuous time switching at low frequency.
Response kernel	Bursting	The decoding methods apply to any response kernel.
	Decaying	
	Delaying	

To represent various methods, we use a unified term

$$\begin{array}{c} \{,\text{i},\text{m}\}\{\text{BF},\text{APF}\}\{,\text{g}\}-\{\text{F},\text{lag},\text{FB}\}. \end{array}$$ (8)

The prefix i or m stands for individual decoding or marginal likelihood decoding in parallel processing, respectively. The main term BF or APF indicates the filtering algorithm. The suffix g stands for using the geometric mean for the likelihood value. Finally, the last part represents whether we use filtering (F), fixed-lag smoothing (lag) or fixed-interval smoothing with the forward-filtering backward-smoothing algorithm (FB). For example, the method iBF-lag represents individual decoding in parallel processing using bootstrap filtering with fixed-lag smoothing.

#### State space model

**Fig 1:**
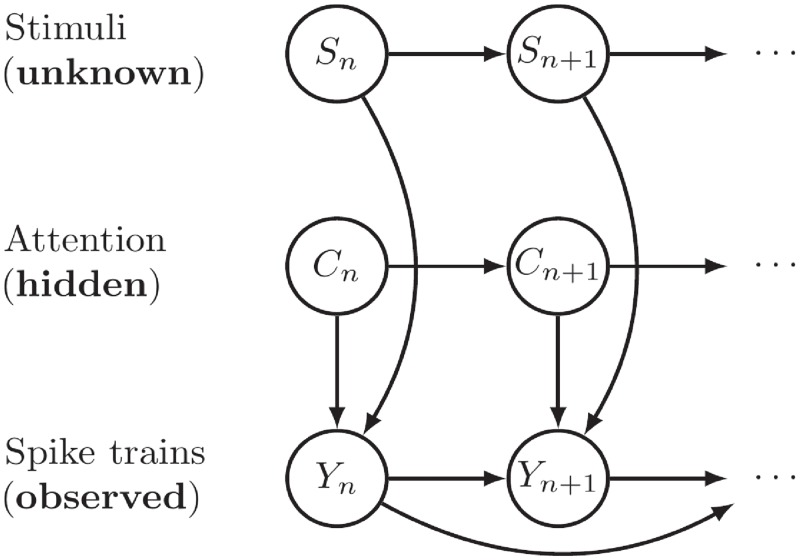
State-space model used for the decoding of stochastic stimuli.

We use a state-space model to describe the evolution of the stochastic stimuli. The state space is extended to not only include the stimuli *S*, but also the unknown stimulus-related parameters, which are included for the construction of the decoding algorithms. Fig 1 shows the graph of the state-space model. The stimuli *S* are continuous-state Markov processes, and the attention states *C* are discrete-state Markov processes. The spike trains *Y* depend on both stimuli and the attention, also affected by spiking history. *S*, *C* and *Y* may be multi-dimensional containing multiple stimuli and neurons. The transition of the states *S* and *C* are parameterized by *θ* = (*γ*, *β*, **Γ**). In the algorithms, the parameters *θ* are also considered as states propagating following Markov processes given in (10), but are not shown in the graph. Denote by *Z*_*n*_ = (*S*_*n*_, *C*_*n*_, *θ*_*n*_) the full hidden states, and *z*_*n*_ a realization of *Z*_*n*_. Similar methods were used in [32], where the authors employed a state-space model describing spike train data with Poisson distributions and an animal’s position with Gaussian noise. Sequential Monte Carlo methods were used to estimate parameters and decode the position based on spike trains. Here we include the latent states explaining visual attention and describe spike trains with leaky integrate-and-fire models. The full states are

$$\begin{array}{c} \begin{array}{cc} \Gamma_{n} & \;(\text{TPM})\\ C_{n} & \;(\text{index}\;\text{of}\;\text{attended}\;\text{stimulus})\\ \gamma_{n} & \;(\text{common}\;\text{diffusion}\;\text{parameter}\;\text{of}\;\text{all}\;\text{stimuli})\\ \beta_{n}=\left(\beta_{n}^{1},\ldots ,\beta_{n}^{K}\right) & \;(\text{drift}\;\text{parameter}\;\text{of}\;\text{each}\;\text{stimulus})\\ S_{n}=\left(S_{n}^{1},\ldots ,S_{n}^{K}\right) & \;(\text{value}\;\text{of}\;\text{each}\;\text{stimulus}) \end{array} \end{array}$$ (9)

The subscript *n* stands for the current time in the state evolution. Note that, even if **Γ**, *γ* and *β* are constant in model (7), the filters will at each time point update the information regarding their value, and thus, they are allowed to change at each time point. Hopefully, they converge towards their true values as more spikes are used in the decoding algorithm. The propagation of states at time *n* is given by:

$$\begin{array}{c} \begin{array}{cccc} {\left\{\lambda_{kl,n}\right\}}_{l=1,\ldots ,K} & ∼\text{Dir}({\left\{V_{\lambda }^{-1}\lambda_{kl,n-1}\right\}}_{l=1,\ldots ,K}); & & \sum_{l=1}^{K}\lambda_{kl,n}=1,\lambda_{kl,n}\geq 0\\ C_{n} & ∼\Gamma (C_{n-1}); & & C_{n}\in \left\{1,\ldots ,K\right\}\\ \gamma_{n} & ∼N_{tr}\left(\gamma_{n-1},V_{\gamma }\right); & & \gamma_{n}>0\\ \beta_{n}^{k} & ∼N(\beta_{n-1},V_{\beta });\\ S_{n}^{k} & ∼N(M_{n}^{k},V_{n}^{k}); \end{array} \end{array}$$ (10)

for *k*, *l* = 1, …, *K*. The state propagation is explained as follows. Each row of the TPM is sampled following a Dirichlet distribution, with parameters being the probabilities in the previous time step multiplied by a concentration parameter $V_{\lambda }^{-1}$ controlling the sampling variance. The index of the attended stimulus is sampled from a multinomial distribution given by row *C*_*n*−1_ of the TPM, **Γ**(*C*_*n*−1_). The parameters *γ*_*n*_ and *β*_*n*_ are updated using Gaussian distributions with variance *V*_*γ*_ and *V*_*β*_, respectively. Since *γ*_*n*_ > 0, a positive truncated Gaussian distribution is used. The strength of each stimulus, $S_{n}^{k}$, is updated according to the OU model, following a Gaussian distribution with mean $M_{n}^{k}=\left(S_{n-1}^{k}-\beta_{n}^{k}\right)e^{-\Delta t}+\beta_{n}^{k}$ and variance $V_{n}^{k}=\gamma_{n}^{2}\left(1-e^{-2\Delta t}\right)/2$.

The likelihood of the spike train given the parameters is obtained from the encoding model. In the following text before we deal with multiple simultaneous spike trains, we focus on decoding of single spike trains, so we will use *y* as a single spike train for readability. Let $y_{n}=\left(t_{1},\ldots ,t_{L_{n}}\right)$ denote the spike train within the duration of the *n*th interval, where it can happen that *y*_*n*_ is empty if no spikes were fired. Since the intervals are short, we need to take into account boundary effects, i.e., the time from the left boundary of the interval to the first spike, and the time from the last spike to the right boundary. Let *T*_*b*_ and *T*_*e*_ denote the beginning and the end of the interval, respectively. Then if *y*_*n*_ is non-empty, $T_{b}\leq t_{1}<\cdots <t_{L_{n}}\leq T_{e}$. Given stimulus *S*_1:*n*_ = *s*_1:*n*_ and attentional index *C*_1:*n*_ = *c*_1:*n*_ from the first to the *n*th time step, the likelihood of *y*_*n*_ is then

$$\begin{array}{c} \begin{array}{cccc} p(y_{n}|s_{n}^{c_{n}},s_{n-1}^{c_{n-1}},H_{T_{b}})= & \prod_{l=2}^{L_{n}}g\left(t_{l}|s_{n}^{c_{n}},H_{t_{l-1}}\right) & & \left(\text{complete}\;\text{ISIs}\;\text{inside}\;\text{the}\;\text{interval}\right)\\ & \times g(t_{1}|s_{n}^{c_{n}},s_{n-1}^{c_{n-1}},H_{T_{b}}) & & \left(\text{left}\;\text{boundary}\right)\\ & \times [1\;-\;\int_{t_{L_{n}}}^{T_{e}}\;g\left(\tau |s_{n}^{c_{n}},H_{t_{L_{n}}}\right)d\tau ] & & \left(\text{survival}\;\text{probability}\;\text{at}\;\text{right}\;\text{boundary}\right) \end{array} \end{array}$$ (11)

If there are no spikes in the interval, the likelihood is given by the survival probability:

$$\begin{array}{c} p\left(y_{n}|s_{n}^{c_{n}},s_{n-1}^{c_{n-1}},H_{T_{b}}\right)=1-\int_{T_{b}}^{T_{e}}g\left(\tau |s_{n}^{c_{n}},s_{n-1}^{c_{n-1}},H_{T_{b}}\right)d\tau . \end{array}$$ (12)

The decoding of stimuli aims at obtaining the conditional distribution

$$\begin{array}{c} p\left(s_{1:n}|y_{1:n}\right)=\int_{\Theta }\sum_{c_{1:n}}^{}p\left(z_{1:n}|y_{1:n}\right)\text{d}\theta =\int_{\Theta }\{\sum_{c_{1:n}}^{}p\left(s_{1:n}|c_{1:n},\theta_{1:n},y_{1:n}\right)p\left(c_{1:n}|\theta_{1:n}\right)p\left(\theta_{1:n}\right)\}\text{d}\theta . \end{array}$$ (13)

We have different types of filtering based on the distribution *p*(*s*_1:*n*_|*y*_1:*n*_).

*Online filtering* refers to the distribution *p*(*s*_1:*n*_|*y*_1:*n*_) or the marginal distribution *p*(*s*_*n*_|*y*_1:*n*_) where *n* represents the current time step. In online filtering, when new data *y*_*n*_ arrive, the unknown hidden state *s*_*n*_ is inferred, and the decoding procedure is online.

*Offline smoothing* refers to the distribution *p*(*s*_1:*N*_|*y*_1:*N*_) or the marginal *p*(*s*_*n*_|*y*_1:*N*_), where *N* represents the total time and *n* is some past time. In offline smoothing, we infer any states in the past *s*_*n*_, *n* = 1, …, *N*, after we observe all data *y*_1:*N*_, and the decoding procedure is offline.

A third type is a *semi-online smoothing*, where we target the distribution *p*(*s*_*n*−Δ*n*_|*y*_1:*n*_), for Δ*n* > 0. We infer the state at a past time *s*_*n*−Δ*n*_ after we receive the data at the current time *y*_1:*n*_. This semi-online decoding procedure can be conducted if we allow for some delay Δ*n* before reporting the online result.

#### A bootstrap particle filter

Sequential Monte Carlo methods aim to obtain the distribution (13) through sequential sampling over time, and the strategy relies on the following decomposition:

$$\begin{array}{c} p\left(z_{1:n}|y_{1:n}\right)=\frac{p(z_{0:n-1}|y_{0:n-1})}{p(y_{n}|y_{0:n-1})}p\left(z_{n}|z_{n-1}\right)p\left(y_{n}|z_{n},y_{0:n-1}\right). \end{array}$$ (14)

The method is to sample a new *z*_*n*_ at each time step *n* and sequentially update the weight of each sample *z*_*n*_ based on the above decomposition [31]. In the bootstrap particle filter (BF), *z*_*n*_ is sampled from *p*(*z*_*n*_|*z*_*n*−1_) and the weight of each sample is updated using *p*(*y*_*n*_|*z*_*n*_, *y*_0:*n*−1_). Each particle is a sample from the state space at all time points, where we write *Z*_*n*,*i*_ = *z*_*n*,*i*_ for the sampled value of *Z*_*n*_ of particle *i*. Particle filtering approximates the distribution *p*(*z*_1:*n*_|*y*_1:*n*_) by the empirical distribution using *I* particles:

$$\begin{array}{c} \hat{p}\left(z_{1:n}|y_{1:n}\right)=\sum_{i=1}^{I}1_{\left\{z_{1:n}=z_{1:n,i}\right\}}{\bar{w}}_{n,i}, \end{array}$$ (15)

where ${\bar{w}}_{n,i}$ denotes the normalized weight of particle *i* at time *n*. Since we are interested only in the marginal distribution of the stimuli, *p*(*s*_1:*n*_|*y*_1:*n*_), we use the marginal

$$\begin{array}{c} \hat{p}\left(s_{1:n}|y_{1:n}\right)=\sum_{i=1}^{I}1_{\left\{s_{1:n}=s_{1:n,i}\right\}}{\bar{w}}_{n,i} \end{array}$$ (16)

and also

$$\begin{array}{c} \hat{p}\left(s_{n}|y_{1:n}\right)=\sum_{i=1}^{I}1_{\left\{s_{n}=s_{n,i}\right\}}{\bar{w}}_{n,i} \end{array}$$ (17)

with the same set of weight values. Then the stimulus at time *n* is estimated by the posterior mean,

$$\begin{array}{cc} {\hat{S}}_{n} & =\sum_{i=1}^{I}s_{n,i}{\bar{w}}_{n,i}. \end{array}$$ (18)

Using the state evolution and the likelihood, the BF is formulated in Algorithm 1. In this particle filter, each particle has the attended target *C*_*n*_ as a state, and only the information about the attended stimulus is used to calculate the weights. In the first step at *n* = 1, the states are initialized by sampling from uniform distributions. The attention state *C* is sampled from a discrete uniform distribution on the indices of the *K* stimuli, *U*{1, …, *K*}, and the other states are sampled from continuous uniform distributions, with intervals given in the Result section.

In this and the subsequent filters, we resample particles using systematic resampling to avoid weight degeneracy, which is conducted as follows. Denote by *U*_*j*_, for *j* = 0, 1, …, *I* − 1, a total of *I* random grid variables. A uniform variable $\bar{U}$ is sampled from *U*(0, 1]. The grid variables follow

$$\begin{array}{c} U_{j}=\frac{j+\bar{U}}{I},\;j=0,1,\ldots ,I-1. \end{array}$$ (19)

The number of duplicates for particle *i*, *i* = 1, 2, …, *I*, after resampling is

$$\begin{array}{c} W_{i}=|\{j;U_{j}\in \left(\sum_{l=1}^{i-1}{\bar{w}}_{l},\sum_{l=1}^{i}{\bar{w}}_{l}\right],j=0,1,\ldots ,I-1\}|, \end{array}$$ (20)

i.e., the number of grid variables that fall into the *i*th increment of the cumulative sum of the normalized weights. It follows that $\sum_{i}^{I}W_{i}=I$ and *W*_*i*_ ≥ 0 for *i* = 1, 2, …, *I*. Afterwards, we set the weight of all resampled particles to 1/*I*.

**Algorithm 1** Bootstrap particle filter, BF

**Initialization:** at *n* = 1

1: **for** particle *i* = 1, …, *I* **do**

2:  Sample each row of **Γ** using the Dirichlet distribution with equal weights

3:  *C*_1,*i*_ ∼ *U*{1, …, *K*}; *γ*_1,*i*_ ∼ *U*(0, max_*γ*_); $\beta_{1,i}^{k}∼U\left(0,\max_{\beta }\right)$; $S_{1,i}^{k}∼U\left(0,\max_{S}\right)$, *k* = 1, …, *K*

4:  Calculate the weights, $w_{i}=p\left(y_{1}|S_{1,i}^{C_{1,i}}\right)$

5: **end for**

6: Calculate normalized weights, ${\bar{w}}_{i}=w_{i}/\sum_{i}w_{i}$

**Iteration:** for *n* = 2, …, *N*

7: Resample particles (systematic resampling)

8: **for** particle *i* = 1, …, *I* **do**

9:  Propagate states: first **Γ**_*n*,*i*_, then *C*_*n*,*i*_, *γ*_*n*,*i*_, *β*_*n*,*i*_, and finally, *S*_*n*,*i*_, from distributions (10)

10:  Calculate the weights, $w_{i}=p\left(y_{n}|S_{n,i}^{C_{n,i}},S_{n-1,i}^{C_{n-1,i}},y_{1:n-1}\right)$

11: **end for**

12: Calculate normalized weights, ${\bar{w}}_{i}=w_{i}/\sum_{i}w_{i}$

13: Estimate attended stimulus, $\hat{S_{n}}=\sum_{i=1}^{I}{\bar{w}}_{i}S_{n,i}^{C_{n,i}}$

#### Auxiliary particle filter with parameter estimation

In the bootstrap filter, the resampling weights are calculated from the past observations. A more reasonable idea is to calculate the weights based on the current observation. In the auxiliary particle filter (APF) [33], the resampling relies on auxiliary variables, for example, the likelihood of the current observation conditional on the expected states:

$$\begin{array}{c} u_{n}=w_{n-1}p\left(y_{n}|\mu_{n}^{C_{n}},S_{n-1}^{C_{n-1}},y_{1:n-1}\right), \end{array}$$ (21)

where

$$\begin{array}{c} \mu_{n}^{C_{n}}=E\left(S_{n}^{C_{n}}|S_{n-1}^{C_{n}},\theta_{n-1}\right). \end{array}$$ (22)

The idea is that the resampling based on the current observation provides particles that are distributed more closely to the posterior at the following time point. Therefore, the weights degenerate less and the effective number of particles is larger.

The stimulus model contains fixed hyperparameters *θ* that are estimated using artificial propagation, which introduces information loss over time [34]. To overcome this, we propagate the hyperparameter *γ*_*n*_ using kernel smoothing as proposed by [34]. The propagation of *γ*_*n*_ follows a Gaussian distribution

$$\gamma_{n+1}∼N(\psi \gamma_{n}+\left(1-\psi \right){\bar{\gamma }}_{n},h^{2}v_{n}),$$ (23)

where ${\bar{\gamma }}_{n}$ and *v*_*n*_ are the mean and the variance of the posterior *p*(*γ*|*y*_1:*n*_), evaluated from particles at time *n*. In practice, we use a truncated version of the Gaussian distribution in (23) since the parameter *γ* is positive. The constants *ψ* = (3*δ* − 1)/2*δ* and *h*^2^ = 1 − *ψ*^2^ are evaluated using a discount factor *δ* ∈ (0, 1], typically around 0.95 − 0.99 recommended by the authors. For the parameters **Γ**_*n*_ and *β*_*n*_, which depend on the stimulus components, we use the same propagation distribution as before, due to the problem of label switching in mixture models [35, 36]. It is difficult to evaluate the posterior of elements of **Γ**_*n*_ and *β*_*n*_ because each particle can label each component differently.

The APF with kernel smoothing of parameters is formulated in Algorithm 2.

**Algorithm 2** Auxiliary particle filter with kernel smoothing, APF

**Initialization:** at *n* = 1

1: **for** particle *i* = 1, …, *I* **do**

2:  Sample each row of **Γ** using the Dirichlet distribution with equal weights

3:  *C*_1,*i*_ ∼ *U*{1, …, *K*}; *γ*_1,*i*_ ∼ *U*(0, max_*γ*_); $\beta_{1,i}^{k}∼U\left(0,\max_{\beta }\right)$; $S_{1,i}^{k}∼U\left(0,\max_{S}\right)$, *k* = 1, …, *K*

4:  Calculate the weights, $w_{i}=p\left(y_{1}|S_{1,i}^{C_{1,i}}\right)$

5: **end for**

**Iteration:** for *n* = 2, …, *N*

6: **for** particle *i* = 1, …, *I* **do**

7:  Propagate **Γ**_*n*,*i*_ and then *C*_*n*,*i*_

8:  Calculate $\mu_{n,i}^{C_{n,i}}=E\left(S_{n,i}^{C_{n,i}}|S_{n-1,i}^{C_{n,i}},\theta_{n-1,i}\right)$

9:  Calculate the first-stage weight, $u_{i}=w_{i}p\left(y_{n}|\mu_{n,i}^{C_{n,i}},S_{n-1,i}^{C_{n-1,i}},y_{1:n-1}\right)$

10: **end for**

11: Resample particles (systematic resampling) using {*u*_*i*_}, giving a new set of particles N

12: **for** particle j∈N **do**

13:  propagate *γ*_*n*,*j*_ using (23), then *β*_*n*,*j*_, and finally *S*_*n*,*j*_

14:  Evaluate the weight, $w_{j}=p\left(y_{n}|S_{n,j}^{C_{n,j}},S_{n-1,j}^{C_{n-1,j}},y_{1:n-1}\right)/p\left(y_{n}|\mu_{n,j}^{C_{n,j}},S_{n-1,j}^{C_{n-1,j}},y_{1:n-1}\right)$

15: **end for**

16: Normalize weights and output estimate

#### Particle filtering with marginal likelihood

In Algorithms 1 and 2 we use the attended target *C* as a hidden state, and the weights are evaluated conditional on *C*. Alternatively, we can marginalize out *C* in each particle, and use all *S* = (*S*^1^, …, *S*^*K*^) to calculate the marginal likelihood as the weight. This requires a recursive computation of the probabilities *p*(*C*_*n*_|*y*_1:*n*−1_, *s*_1:*n*_) at time *n*, for which we follow the routine shown below:

$$\begin{array}{c} p(y_{n}|y_{1:n-1},s_{1:n})=\sum_{j=1}^{K}p\left(y_{n}|C_{n}=j,y_{1:n-1},s_{1:n}\right)p\left(C_{n}=j|y_{1:n-1},s_{1:n}\right); \end{array}$$ (24)

$$\begin{array}{c} p(C_{n}=j|y_{1:n-1},s_{1:n})=\sum_{i=1}^{K}p\left(C_{n-1}=i|y_{1:n-1},s_{1:n-1}\right)\lambda_{ij,n}; \end{array}$$ (25)

$$\begin{array}{c} p(C_{n-1}=i|y_{1:n-1},s_{1:n-1})\propto p\left(y_{n-1}|C_{n-1}=i,y_{1:n-2},s_{1:n-1}\right)p\left(C_{n-1}=i|y_{1:n-2},s_{1:n-1}\right). \end{array}$$ (26)

At time *n*, the probabilities *p*(*C*_*n*_|*y*_1:*n*−1_, *s*_1:*n*_) are computed using *p*(*C*_*n*−1_|*y*_1:*n*−2_, *s*_1:*n*−1_) from time *n* − 1, and likewise in the subsequent time steps. Note that in the marginal probability we depend on all stimuli *S* = (*S*^1^, …, *S*^*K*^) instead of a component given by *C* as in Eq (11).

Due to label switching, each particle could label the stimulus components differently. It is then difficult to output the correct results with the posterior mean [35]. Here we use a simple method. The stimuli in each particle are sorted first, then the posterior mean is calculated for the sorted stimuli. The hope is that after sorting, each particle relabels the components in the same order. The algorithm of a bootstrap particle filter with marginal likelihood is formulated in Algorithm 3.

For single spike trains, we cannot decode all components of the stimulus mixture because only one is attended at a time. Therefore marginal likelihood is less appealing for single spike train decoding. However, if we have multiple independent observations at each time point, marginal likelihood will be more appropriate.

**Algorithm 3** Bootstrap particle filter with marginal likelihood, mBF

**Initialization:** at *n* = 1

1: **for** particle *i* = 1, …, *I* **do**

2:  Sample each row of **Γ** using the Dirichlet distribution with equal weights

3:  *γ*_1,*i*_ ∼ *U*(0, max_*γ*_); $\beta_{1,i}^{k}∼U\left(0,\max_{\beta }\right)$; $S_{1,i}^{k}∼U\left(0,\max_{S}\right)$, *k* = 1, …, *K*

4:  Calculate the weights, *w*_*i*_ = *p*(*y*_1_|*S*_1,*i*_)

5: **end for**

6: Calculate normalized weights, ${\bar{w}}_{i}=w_{i}/\sum_{i}w_{i}$

**Iteration:** for *n* = 2, …, *N*

7: Resample particles (systematic resampling)

8: **for** particle *i* = 1, …, *I* **do**

9:  Propagate states: first **Γ**_*n*,*i*_, then *γ*_*n*,*i*_, *β*_*n*,*i*_ and finally *S*_*n*,*i*_ from distributions (10)

10:  Calculate the weights, *w*_*i*_ = *p*(*y*_*n*_|*S*_*n*,*i*_, *S*_*n*−1,*i*_, *y*_1:*n*−1_)

11: **end for**

12: Calculate normalized weights, ${\bar{w}}_{i}=w_{i}/\sum_{i}w_{i}$

13: Estimate all $S_{n}=\left(S_{n}^{1},\ldots ,S_{n}^{K}\right)$ using $\hat{S_{n}^{k}}=\sum_{i=1}^{N}{\bar{w}}_{i}S_{n,i}^{k}$ on sorted stimulus components

#### Auxiliary particle filtering with parameter estimation and marginal likelihood

The idea of APF and parameter learning using kernel smoothing can also be applied to the particle filter with marginal likelihood. We calculate the first-stage weights using marginal likelihood:

$$\begin{array}{c} u_{n}=w_{n-1}p\left(y_{n}|\mu_{n},S_{n-1},y_{1:n-1}\right), \end{array}$$ (27)

where *μ*_*n*_ is the expectation of all components of *S*_*n*_:

$$\begin{array}{c} \mu_{n}=E\left(S_{n}|S_{n-1},\theta_{n-1}\right). \end{array}$$ (28)

The calculation of the marginal likelihood *p*(*y*_*n*_|*μ*_*n*_) follows the same way as in Eq (24). Due to label switching, only the propagation of the common parameter *γ*_*n*_ is done using the kernel smoothing method by [34]. The algorithm is formulated in Algorithm 4.

**Algorithm 4** Auxiliary particle filter with kernel smoothing and marginal likelihood, mAPF

**Initialization:** at *n* = 1

1: **for** particle *i* = 1, …, *I* **do**

2:  Sample each row of **Γ** using the Dirichlet distribution with equal weights

3:  *γ*_1,*i*_ ∼ *U*(0, max_*γ*_); $\beta_{1,i}^{k}∼U\left(0,\max_{\beta }\right)$; $S_{1,i}^{k}∼U\left(0,\max_{S}\right)$, *k* = 1, …, *K*

4:  Calculate the weights, *w*_*i*_ = *p*(*y*_1_|*S*_1,*i*_)

5: **end for**

**Iteration:** for *n* = 2, …, *N*

6: **for** particle *i* = 1, …, *I* **do**

7:  Calculate $\mu_{n,i}=E\left(S_{n,i}|S_{n-1,i},\theta_{n-1,i}\right)$

8:  Calculate the first-stage weight, *u*_*i*_ = *w*_*i*_*p*(*y*_*n*_|*μ*_*n*,*i*_, *S*_*n*−1,*i*_, *y*_1:*n*−1_)

9: **end for**

10: Resample particles (systematic resampling) using {*u*_*i*_}, giving a new set of particles N

11: **for** particle j∈N **do**

12:  propagate *γ*_*n*,*j*_ using (23), then *β*_*n*,*j*_, **Γ**_*n*,*j*_ and finally *S*_*n*,*j*_

13:  Evaluate the weight, *w*_*j*_ = *p*(*y*_*n*_|*S*_*n*,*j*_, *S*_*n*−1,*j*_, *y*_1:*n*−1_)/*p*(*y*_*n*_|*μ*_*n*,*j*_, *S*_*n*−1,*j*_, *y*_1:*n*−1_)

14: **end for**

15: Normalize weights and output estimate based on sorted stimulus components

#### Decoding from multiple spike trains with serial and parallel processing

Now we consider multiple neurons simultaneously recorded in one trial providing multiple spike trains. Since stochastic stimuli contain inevitable noise and are not reproducible by repetitions in real applications, all estimates of the stimuli depend entirely on the spike trains from one trial. Thus, the attentional behavior of the simultaneously recorded neurons is of great importance for understanding the full information of stimuli.

For multiple, simultaneously recorded spike trains we consider two opposing hypotheses for visual search in neuronal attention, namely the serial and the parallel processing. In serial processing, all stimuli are processed sequentially. The neural interpretation is that all neurons attend to the same stimulus at the same time, and switch to another all together. Therefore, all spike trains would have similar spiking patterns. On the contrary, in parallel processing, stimuli are processed in parallel. Each neuron attends its own stimulus and can switch to another stimulus independently of the other neurons. The spike trains are then distinct from each other.

#### Serial processing

For stimulus decoding using particle methods, serial processing essentially means an increase of the observation size at each time point, making the decoding more accurate. However, it only decodes the attended stimulus at any time, and the data contain no information about the other stimuli at that time point. For *M* spike trains, *y* = {*y*^1^, *y*^2^, …, *y*^*M*^}, the likelihood function with the serial processing assumption within a small interval is then

$$\begin{array}{c} p\left(y_{n}\;|\;S_{n}^{c_{n}},S_{n-1}^{c_{n-1}},y_{1:n-1}\right)=\prod_{m=1}^{M}p\left(y_{n}^{m}|S_{n}^{c_{n}},S_{n-1}^{c_{n-1}},y_{1:n-1}^{m}\right). \end{array}$$ (29)

The right hand side is evaluated using expression (11).

#### Parallel processing

In parallel processing each spike train has its own attended stimulus. Stimulus decoding can then estimate multiple components of the mixture. Each single spike train is decoded independently using Algorithms 1 or 2, which produces estimates of each neuron’s attended stimulus at each time point, and then the results from all spike trains give an empirical distribution of the stimulus mixture at each time point. Then we run cluster analysis at each time point in one-dimensional space based on the estimates of stimuli. Since there are outliers (see the Results section), we apply *k*-medoids clustering [37, 38, chpt. 14] using the square root of Euclidean distance as the dissimilarity measure. The *k*-medoids clustering is preferred over *k*-means because *k*-medoids can be more robust against outliers [38]. Furthermore, the square root of the Euclidean distance puts less weight on extreme outliers than the Euclidean distance. Finally, we use the median of each cluster as the estimate for each component of the stimulus mixture.

Another decoding method for parallel processing is to exploit the marginal likelihood since we have multiple independent observations. Now each particle can decode all stimulus components, and all decoded components will be used for the output estimation. When calculating the weights, we need the likelihood, which is the product of the marginal likelihoods of all spike trains:

$$\begin{array}{c} p\left(y_{n}|S_{n},S_{n-1},y_{1:n-1}\right)=\prod_{m=1}^{M}p\left(y_{n}^{m}|S_{n},S_{n-1},y_{1:n-1}^{m}\right), \end{array}$$ (30)

and the right hand side is evaluated using Eq (24).

#### Adjusting auxiliary variables for large data size

In Algorithms 2 and 4 based on APF for population decoding, the auxiliary variables are calculated using the likelihood, which can take extreme values if the sample size is large, e.g., when the data contain multiple spike trains. The consequence is that only few particles with extreme weight values survive the resampling, reducing the posterior variance and leading to the degeneracy of parameter learning [39, 40]. To slow down the degeneracy, we use the geometric mean of the likelihood value over the number of spike trains, $\tilde{p}\left(y_{n}|\mu_{n},S_{n-1},y_{1:n-1}\right)={\left(\prod_{m=1}^{M}p\left(y_{n}^{m}|\mu_{n},S_{n-1},y_{1:n-1}^{m}\right)\right)}^{1/M}$, when calculating the auxiliary variables in Algorithms 2 and 4.

#### Semi-online smoothing

The above online algorithms return estimates of stimuli by approximating the filtering probability conditional on the observation up to the current time, *p*(*s*_1:*n*_|*y*_1:*n*_). An alternative is offline methods that make use of later observations or the entire data set when estimating the stimuli at a past time point. This posterior is referred to as the smoothing distribution. A full-length smoothing reports the posterior of the stimulus at any time *n* conditional on all observations over 1: *N*, *p*(*s*_*n*_|*y*_1:*N*_), but we can also apply partial smoothing when only certain delays are allowed. Say we need to report the stimulus after a delay of Δ*n* time points, then we can decode the stimulus at time *n* using partial smoothing, *p*(*s*_*n*−Δ*n*_|*y*_1:*n*_). Thus, filtering does real-time online decoding, while smoothing does semi-online decoding with some delay or offline decoding after the full observation. Here we pursue the semi-online decoding allowing a delay of Δ*n* before reporting the stimulus, *p*(*s*_*n*−Δ*n*_|*y*_1:*n*_). Two smoothing methods have been tried, the fixed-lag smoothing and the fixed-interval smoothing [41].

In the fixed-lag smoothing, we simply marginalize the filtering distribution *p*(*s*_1:*n*_|*y*_1:*n*_) for time *n* − Δ*n*:

$$\begin{array}{c} \hat{p}\left(s_{n-\Delta n}|y_{1:n}\right)=\sum_{i=1}^{I}1_{\left\{s_{n-\Delta n}=s_{n-\Delta n,i}\right\}}{\bar{w}}_{n,i}, \end{array}$$ (31)

where the weights are the same as the online filtering weights. Then we estimate the stimulus at time *n* − Δ*n* as

$$\begin{array}{c} {\hat{S}}_{n-\Delta n}=\sum_{i=1}^{I}s_{n-\Delta n,i}{\bar{w}}_{n,i} \end{array}$$ (32)

This requires additional memory to store the history of *S*.

In fixed-interval smoothing we apply the forward-filtering backward-smoothing algorithm, and calculate the smoothing distribution *p*(*s*_*n*−Δ*n*_|*y*_1:*n*_) for the desired time *n* − Δ*n*, instead of using the joint filtering distribution *p*(*s*_1:*n*_|*y*_1:*n*_). The smoothing distribution *p*(*s*_*n*−Δ*n*_|*y*_1:*n*_) is obtained using recursive backward smoothing from *n* after a full forward filtering up to *n* [41]. For the semi-online smoothing at *n* − Δ*n*, we keep the online filtering running. Whenever we receive new data *y*_*n*_, we proceed with the online filtering to obtain *p*(*s*_1:*n*_|*y*_1:*n*_) and go back Δ*n* time steps to obtain the smoothing distribution *p*(*s*_*n*−Δ*n*_|*y*_1:*n*_). See Appendix II: Forward-Filtering Backward-Smoothing for a full description of the forward-filtering backward-smoothing algorithm.

#### Continuous-time switching

All the decoding algorithms assume that neuronal attention is fixed within intervals of duration 100*ms*, and only switches between two intervals. To test how robust the algorithms are when this assumption is violated, we also simulate spike trains with continuous-time switching, i.e., the attentional switching does not need to take place exactly between two intervals. One example is that the switching follows a Poisson process, which is used in the simulations. If this is the case, then decoding with discretization will be less accurate. However, if the switching rate is sufficiently low such that the average inter-switch interval is much longer than the discretized intervals, the Poisson attentional switching is well approximated by the approach based on discretization.

A fixed TPM on discretized time points approximates the Poisson switching model well due to the memoryless property of the Poisson process. However, since the TPM is updated at each time point as latent states, the model is easy to extend to non-Poissonian switching allowing for memory effects by adapting the TPM for a specific model. This is not pursued here.

## Results

**Fig 2:**
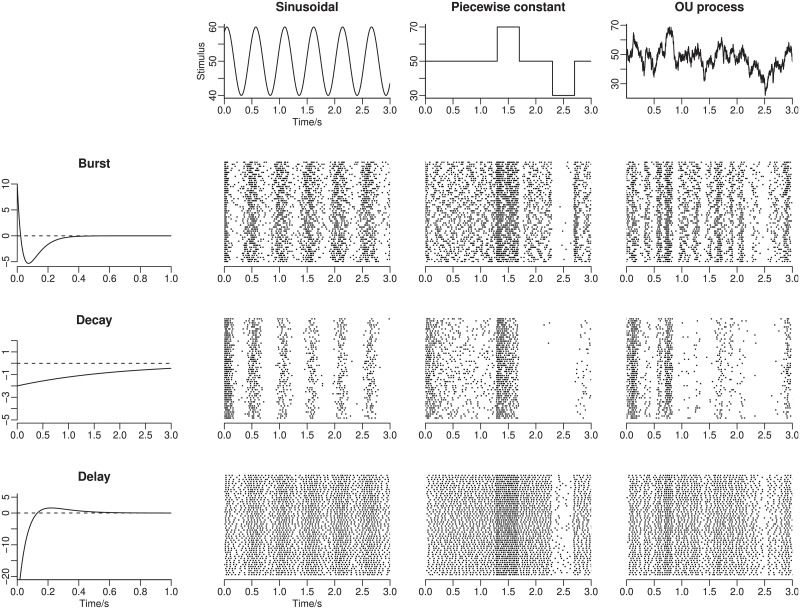
Realizations of spike trains. The left panels show the three response kernels. The top panels show different types of stimuli. Spike trains are shown for each combination of response kernel and stimulus. Each line represents an independent trial. For each combination, 50 example spike trains are simulated.

Throughout the following examples, we use the parameters for the LIF encoding model shown in Table 3. Fig 2 illustrates some realizations of spike trains generated from the encoding model using different response kernels and stimuli.

**Table 3:** Parameters of the LIF encoding model used in the simulations.

Parameter	Value	Explanation
*a*	100	decay rate in LIF model
*x*^−^	0	reflecting boundary of Fokker-Planck equation
*x*_*th*_	1	firing threshold of potential
*x*_0_	0.4	reset potential
*μ*	0.5	resting potential
*σ*	1	diffusion parameter in LIF model
*η*_*burst*_	(50, 25, 40, 15)	burst response kernel
*η*_*decay*_	(0, 0, 2, 0.5)	decay response kernel
*η*_*delay*_	(20, 8, 50, 15)	delay response kernel
Δ*t*	0.002	time discretization in numerical solution
Δ*x*	0.02	potential discretization in numerical solution
Δ*n*	10 intervals	time delay for particle smoothing

In the decoding simulations, we perform many repetition trials. In each decoding trial, we simulate the realizations of the stochastic stimuli and the spike trains, and then perform decoding with the sequential Monte Carlo particle methods. Specifically, we simulate *K* new stimuli according to the OU model. Each spike train is generated using the simulated stimuli within the period [1, 6]s (a period of 5s after 1s burn-in). The time step size of generating the stimulus is 0.01s. We then decode the stochastic mixtures from the spike trains.

The root mean squared deviation (RMSD) between true and decoded stimuli is used to evaluate the performance. Since the stochastic stimuli are simulated with steps of 0.01s and we approximate the stochastic process with a discretized piecewise constant function with steps of 0.1s, we can never achieve a perfect decoding and the RMSD will always be greater than 0. To take this into account, a relative root mean square deviation (rRMSD) is used to measure the decoding accuracy:

$$\begin{array}{c} \text{rRMSD}=\frac{\sqrt{\frac{1}{10N}\sum_{n=1}^{N}\sum_{l=1}^{10}{\left({\hat{S}}_{n}-S_{n,l}\right)}^{2}}}{\sqrt{\frac{1}{10N}\sum_{n=1}^{N}\sum_{l=1}^{10}{\left({\hat{S}}_{n}^{*}-S_{n,l}\right)}^{2}}}. \end{array}$$ (33)

where *N* is the number of discretized intervals, $l=1,2,\ldots ,\frac{0.1s}{0.01s}$ is an index for discretization in each time step, *S*_*n*,*l*_ denotes the true stimulus, different for each *n* and *l*, ${\hat{S}}_{n}$ is the prediction of the stimulus and ${\hat{S}}_{n}^{*}$ is an artificial stimulus that minimizes the RMSD, ${\hat{S}}_{n}^{*}=\frac{1}{10}\sum_{l=1}^{10}S_{n,l}$. Then the best achievable value of rRMSD is 1.

The effective sample size (ESS) measures the weight degeneracy of the sequential Monte Carlo methods. The ESS at time *n* for *I* particles is given by

$$\begin{array}{c} {\left(N_{eff}\right)}_{n}=\frac{1}{\sum_{i=1}^{I}{\left({\bar{w}}_{n,i}\right)}^{2}}. \end{array}$$ (34)

If the weights are evenly spread, then (*N*_*eff*_)_*n*_ = *I* takes its maximum value. The smaller ESS is, the less effective are the particles in representing the distribution.

The performance of different particle methods are compared using rRMSD, ESS and the trace of parameter learning over time.

We tried stimulus mixtures of *K* = 1, 2 and 3 components. A mixture of 1 component implies that the neuron’s attention is fixed at the single stimulus. We set the TPM for the mixture of two or three to

$$\begin{array}{c} \Gamma_{2}=[\begin{array}{cc} 0.8 & 0.2\\ 0.2 & 0.8 \end{array}],\;\Gamma_{3}=[\begin{array}{ccc} 0.5 & 0.2 & 0.3\\ 0.3 & 0.5 & 0.2\\ 0.2 & 0.3 & 0.5 \end{array}]. \end{array}$$ (35)

Table 4 shows the *β* parameters used for each component and the common *γ* values for each mixture.

**Table 4:** Stimulus parameters, *β* and *γ*, of the stochastic stimulus mixtures using OU processes.

Mixture number	one	two		three		
Stimulus index	1	1	2	1	2	3
*β*	70	65	75	60	70	80
*γ*	20	20		20		

During initialization, the values of *γ*, *β* and the stimulus strength *S* are uniformly sampled from *U*(0, 40), *U*(0, 200) and *U*(0, 200), respectively. The parameters for the algorithmic updating of Γ, *γ* and *β* are *V*_λ_ = 0.02, *V*_*γ*_ = 1 and *V*_*β*_ = 4, respectively. For the AFP algorithm with kernel smoothing, we use *δ* = 0.95. Throughout the experiments, the number of particles is *I* = 500. The delay time for particle smoothing is Δ*n* = 10 intervals equal to 1s.

All data are simulated according to the state-space model and the diffusion process described in the Models and Methods section using the parameters given above.

In Table 5 we show a summary of the performance comparison of different methods from the simulations. In both single and multiple spike train simulations, we focus on discrete-time switching and the bursting kernel to compare between different particle algorithms. Then we include extensions with continuous-time switching and other response kernels. The detailed explanations of the results can be found in the following sections. The source code for performing these experiments is in the repository https://osf.io/tkvhs/ (DOI: 10.17605/OSF.IO/TKVHS).

**Table 5:** Summary of results. The signs ≈, < and > denote decoding performance comparison in different settings.

**Single spike trains**		
	Methods	Performance comparison
	BF, APF	fewer stimuli > more stimuli
		APF ≈ BF for fewer stimuli
		APF > BF for more stimuli
		Smoothing > Filtering
**Multiple spike trains**		
	Methods	Performance comparison
Serial (K = 2)	BF, APF, APFg	Multiple spike trains in serial > single spike trains
		APF < BF
		Smoothing ≤ Filtering
		APFg ≈ APF
Parallel (K = 2)	iBF, iAPF, mBF, mAPF, mAPFg	APF ≥ BF
		Smoothing ≤ Filtering for m-
		Smoothing ≥ Filtering for i-
		APFg ≈ APF
**Extensions**		
	Methods	Comments
Continuous-time switching	Poisson process switching	Decoding at switching point may be unstable. Overall performance is close to discrete switching.
Response kernels	Delaying	Delaying ≈ Bursting
	Decaying	Decaying < Bursting, due to low firing rate

### Single spike trains

In single spike train experiments, the decoding trials are repeated 50 times. In each trial new stimuli are generated and one spike train is simulated following the stimulus mixture. Then all decoding is conducted only on this single spike train.

**Fig 3:**
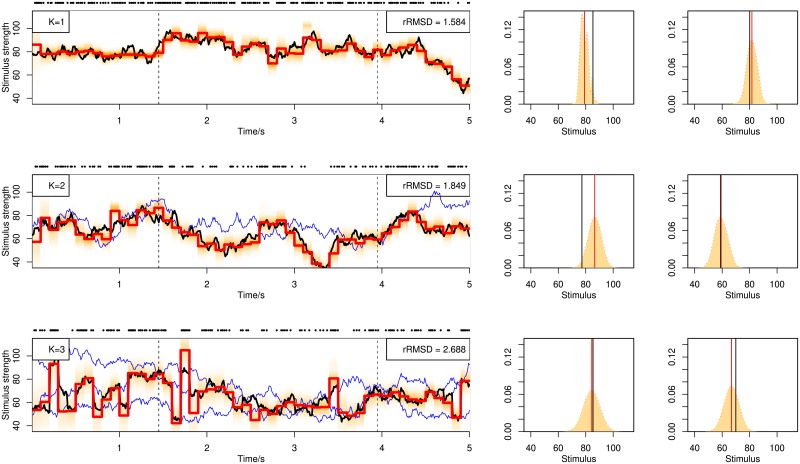
Decoding of stochastic stimulus mixtures using BF with filtering from a single spike train responding to stimulus mixtures containing 1 (upper panel), 2 (middle panel) or 3 (lower panel) components. Blue curves show all the stimulus components in the mixture, and the black curve switching between the blue curves indicate the attended stimulus. Red piecewise-constant lines show the decoding results as the posterior mean, with each constant interval being 100*ms* long. The light red shaded area indicates the posterior distribution at each time step. The spike train is plotted above each decoding figure as sequences of dots. The rRMSD values are shown on the top-right corner of each figure. In the right side of each panel, the empirical posterior distributions at selected time points indicated by dashed lines in the left panels are shown, computed from weighted kernel density smoothing using the particles. The red vertical line indicates the posterior mean, i.e., the decoding estimates shown in the left panels. The black vertical line indicates the true stimulus averaged across the 100*ms* interval.

**Fig 4:**
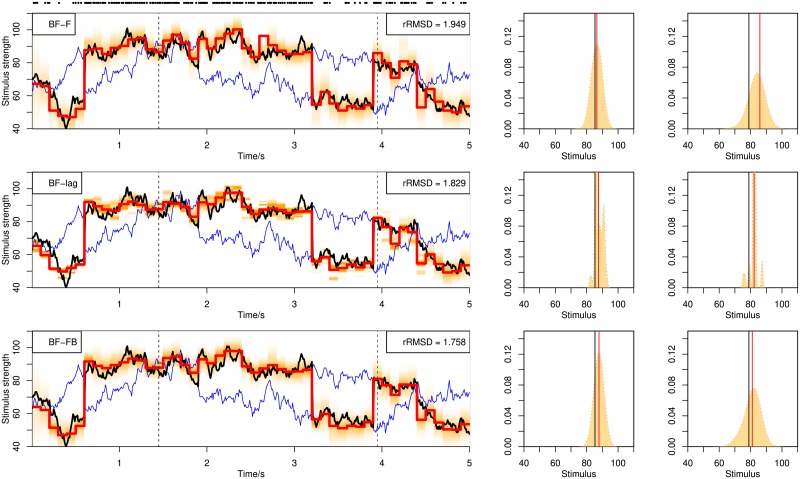
Decoding of stochastic stimulus mixtures from a single spike train. Decoding by BF with filtering, BF-F (upper panel), fixed-lag smoothing, BF-lag (middle panel) and fixed-interval smoothing, BF-FB (lower panel). The three panels show the decoding of the same spike train. See caption of Fig 3 for explanation.

Fig 3 illustrates decoding examples for single spike trains using the online BF. Shown in the figure are single spike trains and the corresponding decoding results (left) together with more detailed illustration of the posterior distributions (middle and right) at selected time points (dashed lines in left figures), using stochastic mixtures of 1, 2 and 3 components in the upper, middle and lower row panels. The posterior distribution (shaded area) is computed from weighted kernel density smoothing using particles. In Fig 4 are shown decoding examples for two stimuli, using online filtering, fixed-lag smoothing and fixed-interval smoothing with a delay of Δ*n* = 10 for the upper, middle and lower row panels. The same spike train is used for the three methods.

**Fig 5:**
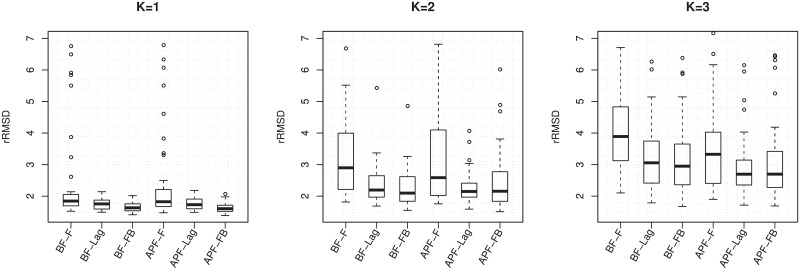
The rRMSD values of decoding stochastic mixtures with *K* = 1, 2 and 3 components using different particle methods, calculated from 50 repetitions. In the labels of the *x*-axis, F: filtering, Lag: fixed-lag smoothing, FB: fixed-interval smoothing using the forward-filtering backward-smoothing algorithm. For example, APF-Lag means using APF and reporting estimates using fixed-lag smoothing.

Boxplots of rRMSD values from 50 repetitions are shown in Fig 5. Various combinations of three filtering methods (online filtering, fixed-lag smoothing and fixed-interval smoothing), two particle methods (BF and APF) and three component sizes (*K* = 1, 2 and 3) are tried. The decoding performance tends to be better when there are less number of stimulus components and when we use delayed smoothing rather than online filtering. The benefit of APF is not observed for *K* = 1 and *K* = 2, but becomes notable when *K* = 3.

**Fig 6:**
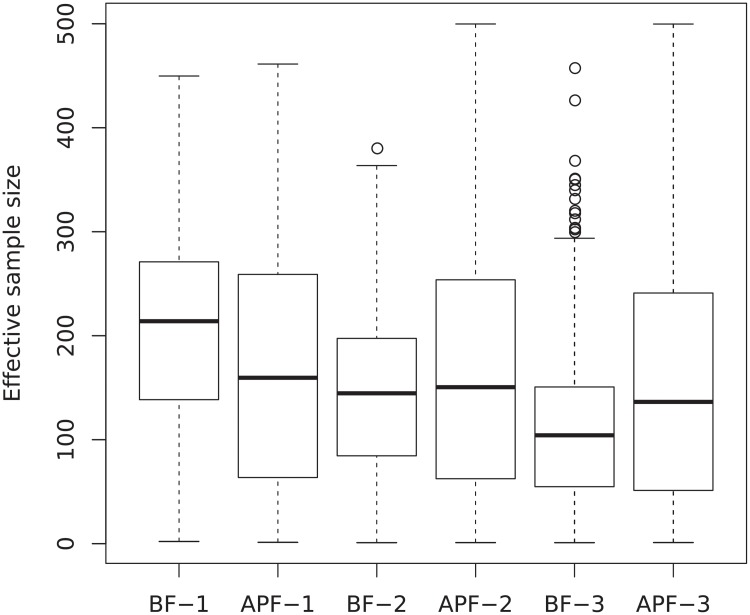
Effective sample sizes. ESS of BF and APF with *K* = 1, 2, 3 stimuli, shown in boxplots for 2500 samples of 50 repetitions at 50 time steps. The labels in the *x*-axis show the number of stimuli. For example, APF-2 means using APF with 2 stimuli.

Fig 6 shows the ESS of different particle methods for different number of components. The ESS is calculated for all time steps, so the boxplots cover 2500 samples for all 50 repetitions at all 50 time steps. The ESS of APF outperforms BF only when *K* = 3. When *K* = 2, the medians of APF and BF are comparable but the variance of BF is smaller. When *K* gets larger, the weight degeneracy quickly becomes a problem for BF, but the weights are less sensitive to *K* for APF. This finding here corresponds to the finding in the rRMSD plots in Fig 5.

**Fig 7:**
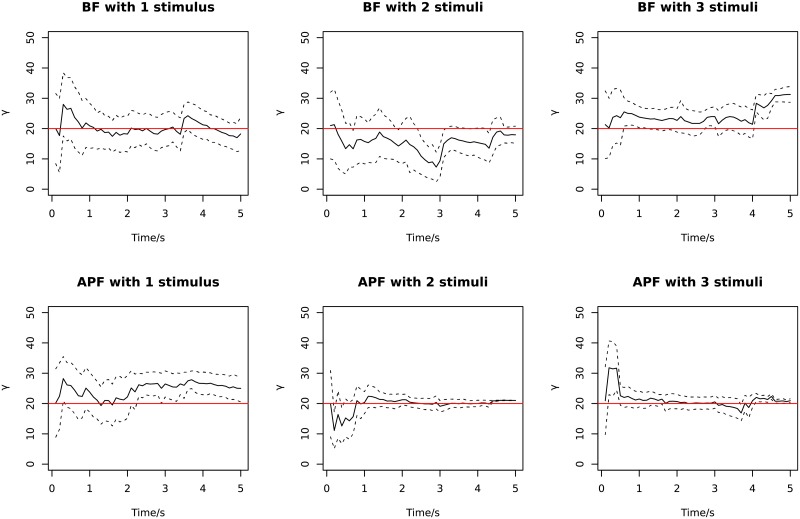
Examples of parameter learning of *γ* over time. The solid line is the mean of 500 particles, and dashed lines show ± the standard deviation. The red lines are the true values.

Finally, in Fig 7 we show examples of the time trajectory of parameter learning for *γ*, the diffusion parameter in the OU model of the stimuli. Parameter learning converges faster using APF than BF when there is more than one stimulus, but the learning is not as fast as the parameter degeneracy (observed and explained in the following population decoding).

### Multiple spike trains

In population decoding of multiple spike trains, we use a mixture of two stimuli also of length 5 *s*. In each trial we simulate new stimuli and 20 simultaneous spike trains, and we conduct 50 repetitions. Population decoding assumes either serial processing or parallel processing.

**Fig 8:**
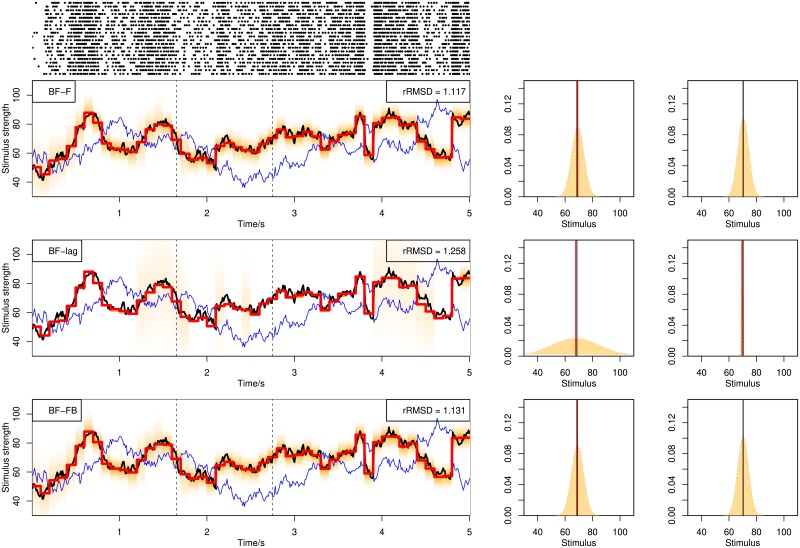
Decoding from 20 spike trains on a stimulus mixture with two components assuming serial processing. Decoding is done by BF with online filtering (upper middle panel), fixed-lag smoothing (lower middle panel) and fixed-interval smoothing (lower panel).

A decoding example following serial processing is shown in Fig 8. The figure compares filtering, fixed-lag smoothing and fixed-interval smoothing, all using BF. In the top of the figure are shown the 20 spike trains used for decoding, which follow similar spiking patterns because all of them attend to the same stimulus assuming serial processing.

**Fig 9:**
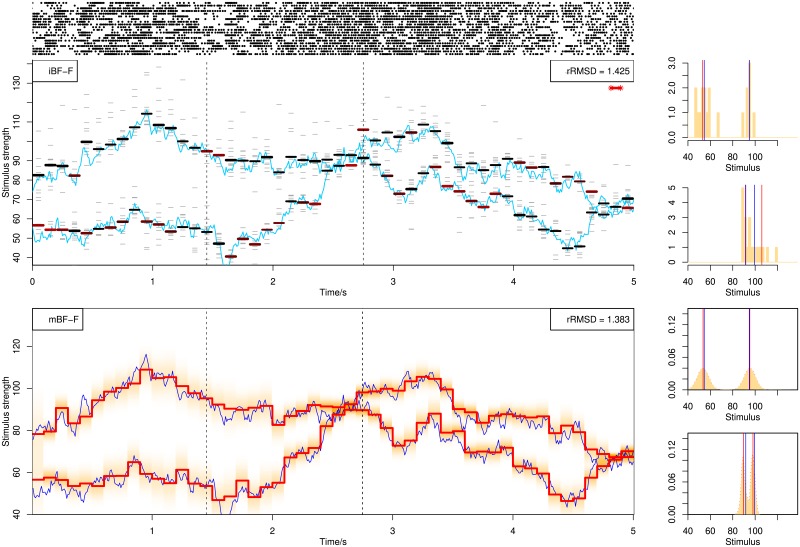
Decoding from 20 spike trains using BF assuming parallel processing. In the top panel 20 spike trains are shown. In the middle panel is shown the method using individual decoding and clustering. Short gray bars show the individual decoding results of stimulus at each time point from 20 spike trains. Thick bars show the medians of clustered categories. A more red color of the thick bars means less number of estimates inside the corresponding category. We mark by two stars if there are less than or equal to 5% estimates in a category (in this case, 5% × 20 = 1 estimates, which only happens once, at time 4.9 *s*). Blue curves show the true stimuli. The histograms to the right show the distribution of 20 estimates with red lines indicating the medians. In the lower panel is shown BF with marginal likelihood. For graphical reasons, we plot the two dimensional posterior estimation of the two stimuli in one dimension. For both decoding methods assuming parallel processing, all stimulus components are decoded at each time point. Blue curves show the true stimuli.

A decoding example following parallel processing is shown in Fig 9. Spike trains can be quite distinct due to different attended stimuli. All stimuli can be simultaneously decoded at each time point. Two decoding methods are used. First we apply individual decoding of each spike train, obtaining 20 estimates which are clustered into two categories. The median of each category is the final estimate. The histograms to the right show the distributions of the 20 estimates at two selected time points. Sometimes one category contains few estimates. This occurs when the two components are different in strength and most spike trains happen to attend to one stimulus component, or when the two components have similar strength and outliers form a second category. A category with few estimates is marked by a red color and stars if ≤5% of the total size. Starred estimates should be ignored to avoid the effect of outliers and the other category will be used as the decoding result for both components. The stars at 4.9 *s* in the middle panel captures a situation where the two stimuli are close. The second method for parallel population decoding is to use marginal likelihood. All stimulus components are decoded due to multiple independent observations at each time point, shown in the lower panel.

**Fig 10:**
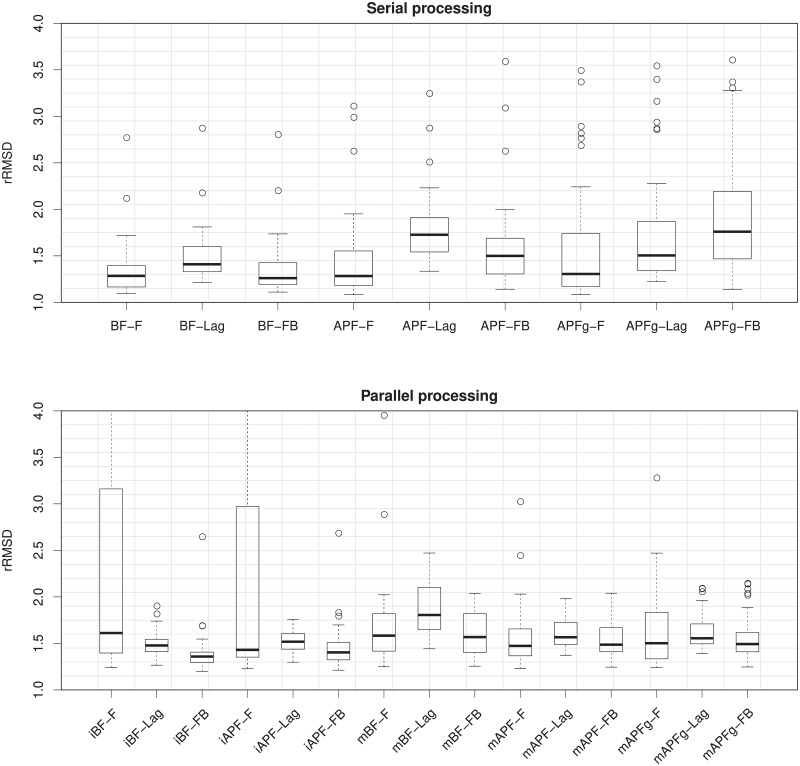
The rRMSD values using different particle methods for serial and parallel processing, calculated from 50 repetitions. In the labels of the *x*-axis, APFg: APF with geometric mean, iBF: individual decoding using BF, iAPF: individual decoding using APF, mBF: BF with marginal likelihood, mAPF: APF with marginal likelihood, mAFPg: APF with marginal likelihood and geometric mean. For example, APFg-FB means using APF with geometric mean, and reporting estimates using fixed-interval smoothing by the forward-filtering backward-smoothing algorithm.

**Fig 11:**
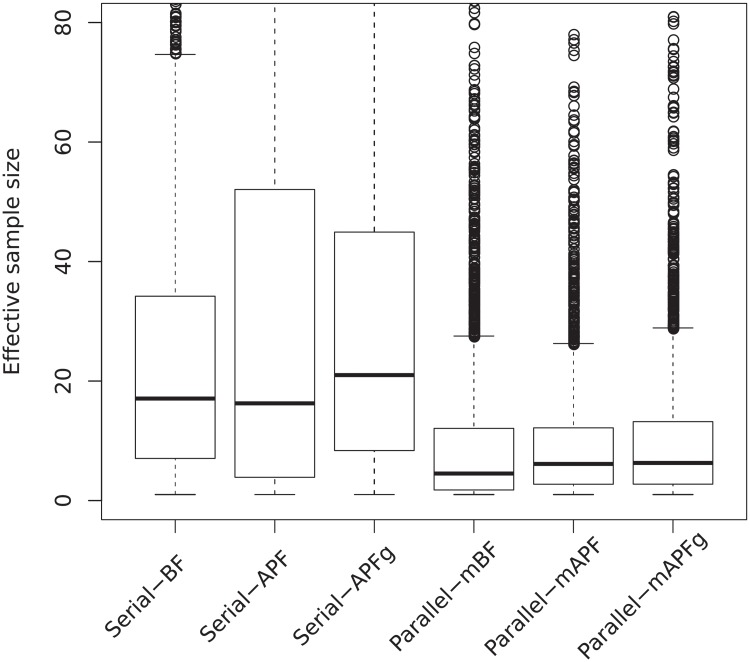
ESS using different methods in serial and parallel processing, shown in boxplots for 2500 samples of 50 repetitions at 50 time steps. The labels in the *x*-axis show the methods used. For example, parallel-mAPFg means using mAPF with geometric mean for parallel processing.

**Fig 12:**
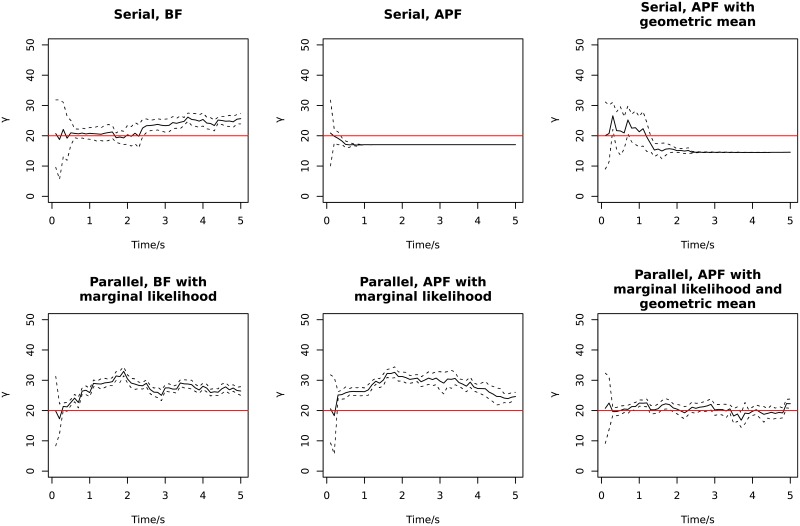
Examples of parameter learning of *γ* over time. The solid line is the mean of 500 particles, and dashed lines show ± the standard deviation. The red lines are the true value.

In Fig 10 the rRMSD from 50 repetitions of different methods are shown as boxplots. Population decoding using multiple spike trains generally performs better than single spike train decoding. For serial processing, APF performs worse than BF, and for parallel processing APF performs as well as or better than BF, judging from the rRMSD results. For both serial and parallel population processing methods, smoothing yields little or no improvement over filtering. However, the exception is the individual decoding methods for parallel processing, of course, since they are based on decoding of single stimuli. Indeed, significant improvement is observed when using smoothing instead of filtering for iBF and iAPF. The reason for the performances of BF and APF, filtering and smoothing can be partly found from the ESS values shown in Fig 11. Most notably, the ESS values are much smaller than the ESS values of single spike train results (Fig 6), due to extreme weights for larger sample sizes. This can lead to inaccurate approximations of the marginalization in fixed-lag smoothing and the integrals in the forward-filtering backward-smoothing algorithm. The smoothing performance is more affected by the small ESS than filtering. Furthermore, for serial processing BF has better ESS with higher median and smaller variance than APF, whereas for parallel processing, APF has better ESS. This explains the different performances of BF and APF in serial and parallel processing in Fig 10. Finally, regarding using geometric means, we do not observe much improvement of APFg and mAFPg over APF and mAPF. Using geometric means have positive effects since the ESS’s are larger and the parameter degeneracy slows down (Fig 12) with APFg and mAFPg. However, the geometric mean changes the resulting posterior distribution and introduces a bias.

In Fig 12, examples of parameter learning of *γ* are plotted for different methods. The APF algorithm for serial population decoding suffers from parameter degeneracy. Parameter degeneracy of APF with kernel smoothing [34] under large sample sizes has been reported in previous studies [39], which is a phenomenon where the parameter distribution quickly becomes narrow or collapses to a Dirac delta function. If parameter learning degenerates too fast before it receives sufficient data to achieve a good estimate, the parameter can be fixed at values far from the true one, reducing the decoding accuracy. Using the geometric mean slows down the degeneracy for serial processing. Other parameter learning methods have previously been studied using sufficient statistics, which may avoid the degeneracy problem [39, 40]; it is not pursued here. For particle filtering with marginal likelihood on parallel population decoding, there is not a large difference between APF and BF in terms of degeneracy.

### Approximating continuous-time switching

**Fig 13:**
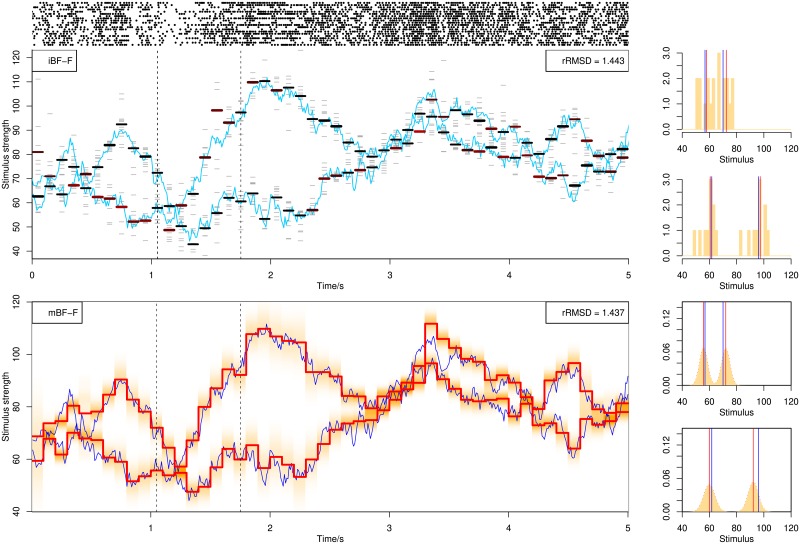
Decoding from 20 spike trains using BF assuming parallel processing. In each spike train, neuronal attention switches at continuous times following a Poisson process.

**Fig 14:**
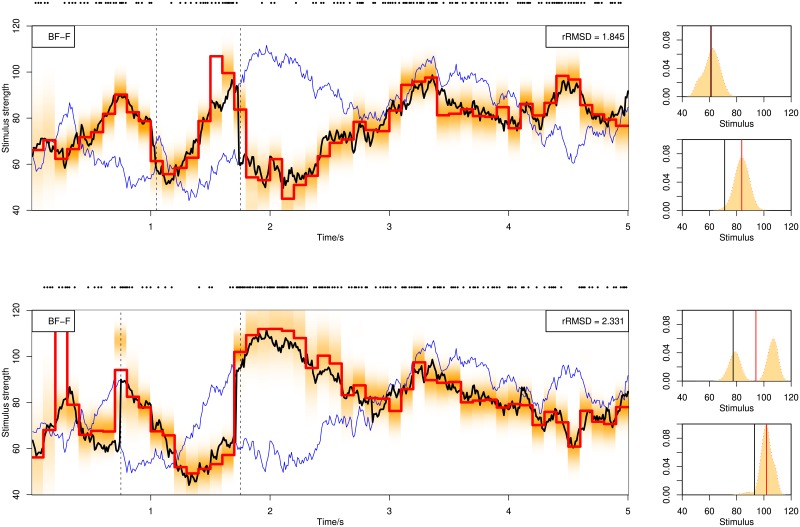
Decoding of two example single spike trains selected from Fig 13 using BF. Neuronal attention switches at continuous times following a Poisson process. Example switching times are indicated by dashed lines.

Here we simulate the attentional switching in continuous time following a Poisson process to test how robust the methods are to discretization errors. With the same setup and methods as above, we conduct the population decoding with parallel processing. In Fig 13 is shown the decoding result of parallel population decoding, and in Fig 14 are shown two examples of single spike train decoding selected from the 20 spike trains in Fig 13. The posterior distribution to the right are taken from the switching time indicated by dashed lines. With a low Poisson switching rate, the decoding accuracy is not severely affected for parallel population decoding. For single spike train decoding, the estimate at switching times tends to be somewhere between the two values before and after the switch (first spike train in the upper panel in Fig 14), but sometimes the estimation can be far from the true stimulus (second spike train at 0.8 *s* in the lower panel in Fig 14).

### Decoding with the delay and decay kernel

**Fig 15:**
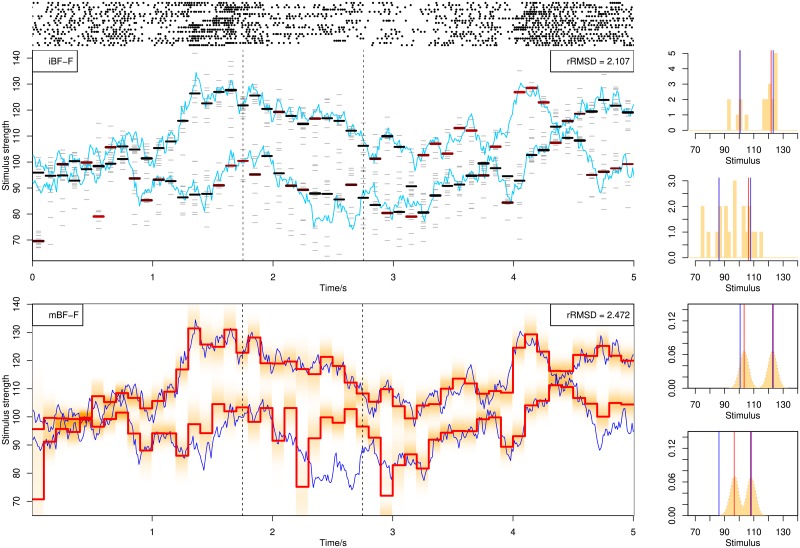
Decoding from 20 spike trains using BF assuming parallel processing, using the decay response kernel.

**Fig 16:**
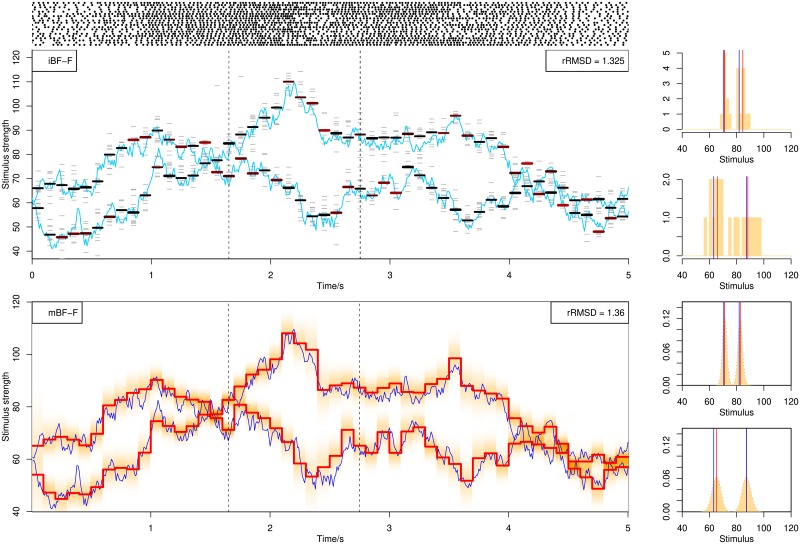
Decoding from 20 spike trains using BF assuming parallel processing, using the delay response kernel.

In the above analysis, we have been using the burst response kernel which generates rhythmic and oscillatory bursting spiking patterns. Now we also try parallel population decoding using the decay and the delay kernel, shown in Figs 15 and 16, respectively. Again we use the same setup and methods. For the delay kernel, good performance is achieved, comparable with the burst kernel. For our current specification of the decay kernel, the spiking rate decreases rapidly over time and we have to use stronger stimulus, but there are still long ISIs (e.g. in the middle region from 2s to 4s) which reduce the decoding accuracy.

## Discussion

We have shown how to decode mixtures of multiple stochastic stimuli in the framework of visual attention under the hypothesis of probability mixing, which assumes the neuron responds to only one single stimulus at any time. The opposing hypothesis is response averaging [42], which assumes the neuron responds to a weighted average of the mixture. In this case, the decoding of each single stimulus would be much harder or impossible due to the difficulty in identifying each single stimulus based on the estimate of the weighted average, and information of individual stimulus characteristics would not be identifiable. This is an argument for why the neural system probably follows the probability-mixing hypothesis, as also shown in [3].

In the decoding simulations with stochastic mixtures, we successfully decode the attended stimulus component using a single spike train or using population data under serial processing. Using population data under parallel processing enables us to obtain information of all stimulus components. Various types of particle methods are employed and compared. Interestingly, we find that the more complicated techniques using APF and kernel-based parameter learning do not necessarily perform better than basic methods using BF, and smoothing, conditional on more observations, does not necessarily perform better than filtering. This is related to sample size and model complexity.

For a limited number of particles (500 in our case), smoothing performance is closely related to ESS and how extreme the weights are. If the sample size is increased, weights become extreme and ESS decreases. After Δ*n* = 10 times of resampling, the values $\left\{S_{n-\Delta n}^{k};k=1,\ldots ,K\right\}$ used in fixed-lag smoothing only contain very few or only one unique value, so the accuracy will be affected. The forward-filtering backward-smoothing algorithm is also affected because the backward sweep requires the integration using the past particles. Therefore, for a large sample size smoothing can perform worse than filtering. In addition, the backward-smoothing procedure requires the transition probabilities *p*(*z*_*n*_|*z*_*n*−1_) that we compute using different particles at time *n* and *n* − 1, and the performance is affected by label switching.

The performance of APF compared with BF has previously been studied; see e.g. [43–45]. APF applies new proposal weights to resample particles by an early introduction of subsequent distributions, as a variance reduction approach: the estimation variance is reduced if we achieve a good prediction of subsequent weights and thus larger ESS. When the data size is large, distributions become narrow and the first-stage weight in APF may not provide a good prediction of the subsequent distribution; meanwhile, the more complicated two-stage numerical calculations under a limited particle size could yield more variance and bias. Therefore, the variance reduction can perform worse for a large data size. When the model is more complicated, so are the prior and transition distributions of the states. It becomes difficult for BF to have good samples with a limited number of particles. APF, on the other hand, gains advantage by introducing the subsequent states information, and therefore suffers less from the increased model complexity than BF. Increased model complexity also makes the distributions less narrow under a large sample size due to higher dimensions. In summary, APF is more favored for smaller sample sizes and more complex models. In our case, population decoding contains a larger sample size than single spike train decoding. Increasing the stimulus number *K* yields higher dimensions and thus a more complex model. With the same *K*, parallel processing with mAPF (using full stimulus information) has larger dimensions than serial processing with APF (using partial stimulus information).

In the simulations of parallel processing, the stimulus number *K* is much less than the number of simultaneously recorded spike trains, and each stimulus component has sufficiently large probability to be attended. Consequently, at all time points each component is likely to be attended by some neurons and it becomes possible to decode all stimulus components. If, on the other hand, *K* is too large, or the probability of attending to one of more components is very small, the decoded stimuli will not likely form as many as *K* clusters. In that case we could try out different *K* values for the clustering analysis, and report the k* which minimizes the Bayesian information criterion. This means that among all *K* stimuli, k* are most likely attended by the recorded neurons and we decode those k* attended stimuli.

We have included some extensions, namely approximating continuous-time switching and various response kernels. The framework can be further extended in much broader ways. For example, we may consider other spiking neuron models like point processes, and spiking models incorporating neuronal interactions, or even other more sophisticated ion channel models if we have access to intracellularly recorded membrane voltage data. This amounts to modifying the likelihood of the observed data conditional on the stimulus and historical data in Eq (6). Another feature of this state-space framework is that we take into account the hidden attentional states, which is particularly useful if we have prior knowledge about neuronal attention. Using prior information, we can e.g. put constraints on the TPM of attention switching, or set appropriate discretization intervals.

Our methods provide a state-space, Monte Carlo framework for neural decoding incorporating single neurons’ attention, which can be easily extended for different neural models and experimental settings. The framework is especially useful for applications with complex stochastic stimuli and multiple simultaneously recorded neurons, or when we want to infer the neuronal attention scheme in addition to decoding the stimuli. The simulation results can serve as a reference to choose proper algorithms when researchers apply the methods to experimental data.

## Appendices

### Appendix I: Probability of ISIs

Suppose the membrane potential *x* resets to *x*_0_ at time 0, and the spike time is *t* > 0. We use the following notation:

$$\begin{array}{c} f(x,t|S,H_{t-})\;(\text{time-evolving}\;\text{probability}\;\text{density}\;\text{function}\;\text{of}\;\text{voltage}) \end{array}$$

$$\begin{array}{c} F(x,t|S,H_{t-})\;(\text{time-evolving}\;\text{cumulative}\;\text{distribution}\;\text{function}\;\text{of}\;\text{voltage}) \end{array}$$

$$\begin{array}{c} g(t|S,H_{t-})\;(\text{probability}\;\text{density}\;\text{function}\;\text{of}\;\text{spiking}\;\text{at}\;t\text{,}\;\text{i.e.,}\;\text{PDF}\;\text{of}\;\text{the}\;\text{ISI}) \end{array}$$

$$\begin{array}{c} G(t|S,H_{t-})\;(\text{cumulative}\;\text{distribution}\;\text{function}\;\text{of}\;\text{spiking}\;\text{at}\;t\text{,}\;\text{i.e.,}\;\text{CDF}\;\text{of}\;\text{the}\;\text{ISI}) \end{array}$$

All the above probabilities depend on the stimulus *S* and the spike history up to the previous spike, $H_{t-}$. In the following, we suppress *S* and $H_{t-}$ in the notation for readability.

The probability that the neuron has not yet fired at time *t*, 1 − *G*(*t*), is equivalent to the probability that the potential has not yet reached *x*_*th*_, *F*(*X*_*th*_, *t*). Thus, the probability density of an ISI is

$$\begin{array}{c} g\left(t\right)=-\frac{\partial }{\partial t}F\left(x_{th},t\right)=-\frac{\partial }{\partial t}\int_{-\infty }^{x_{th}}f\left(x^{\prime },t\right)dx^{\prime }. \end{array}$$ (36)

The transition probability density with a resetting threshold follows the Fokker-Planck equation, defined by the following partial differential equation (PDE):

$$\begin{array}{c} \partial_{t}f\left(x,t\right)=-\partial_{x}\left(b\left(x,t\right)f\left(x,t\right)\right)+\frac{\sigma^{2}}{2}\partial_{xx}^{2}f\left(x,t\right), \end{array}$$ (37)

with absorbing boundary condition *f*(*x*_*th*_, *t*) = 0 and initial condition *f*(*x*, 0) = *δ*(*x* − *x*_0_). For numerical reasons, we also approximate by setting a reflecting boundary condition at a small value *x* = *x*^−^, where the flux equals 0.

Now we formulate a PDE based on the CDF, *F*(*x*, *t*) [22, 46, 47]. Plugging *f*(*x*, *t*) = ∂_*x*_*F*(*x*, *t*) into (37) gives

$$\begin{array}{c} \partial_{t}\partial_{x}F\left(x,t\right)=-\partial_{x}[b\left(x,t\right)\partial_{x}F\left(x,t\right)-\frac{\sigma^{2}}{2}\partial_{x}\partial_{x}F\left(x,t\right)]. \end{array}$$ (38)

Integrating both sides with respect to *x* yields

$$\begin{array}{c} \partial_{t}F\left(x,t\right)=-b\left(x,t\right)\partial_{x}F\left(x,t\right)+\frac{\sigma^{2}}{2}\partial_{xx}^{2}F\left(x,t\right)+C\left(t\right). \end{array}$$ (39)

At the lower reflecting boundary *x* = *x*^−^, we have *F*(*x*^−^, *t*) = 0 and thus ∂_*t*_*F*(*x*, *t*)|_*x* = *x*^−^_ = 0. The flux equals 0, so

$$\begin{array}{cc} J(x^{-},t) & =-b\left(x^{-},t\right)f\left(x^{-},t\right)+\frac{\sigma^{2}}{2}\partial_{x}f\left(x,t\right){|}_{x=x^{-}}\\ & =-b\left(x,t\right)\partial_{x}F\left(x,t\right){{|}_{x=x^{-}}+\frac{\sigma^{2}}{2}\partial_{xx}^{2}F\left(x,t\right)|}_{x=x^{-}}\\ & =0. \end{array}$$ (40)

Thus, *C*(*t*) = 0, and we obtain the PDE for *F*(*x*, *t*):

$$\begin{array}{c} \partial_{t}F\left(x,t\right)=-b\left(x,t\right)\partial_{x}F\left(x,t\right)+\frac{\sigma^{2}}{2}\partial_{xx}^{2}F\left(x,t\right), \end{array}$$ (41)

with boundary conditions ∂_*x*_*F*(*x*_*th*_, *t*) = 0, *F*(*x*^−^, *t*) = 0, and initial condition *F*(*x*, 0) = *H*(*x* − *x*_0_), where *H*(⋅) is the Heaviside step function.

The PDE is solved numerically using Crank-Nicholson finite difference method by discretizing time and potential with grid size Δ*t* and Δ*x*. The result is the CDF *F*(*x*, *t*), which is differentiated along time to obtain the desired *g*(*t*) following Eq (36).

### Appendix II: Forward-filtering backward-smoothing

In the model, the hidden Markov processes are denoted by *Z*_1:*n*_ and the observations by *Y*_1:*n*_. Suppose we have observations up to time *N*, *y*_1:*N*_, and are interested in the smoothing distribution at time *n* < *N*, *p*(*z*_*n*_|*y*_1:*N*_). The smoothing distribution can be decomposed using

$$\begin{array}{cc} & \;p(z_{n}|y_{1:N})\\ =\; & \;p(z_{n}|y_{1:n},y_{n+1:N})\\ =\; & \;\frac{p\left(y_{n+1:N}|z_{n},y_{1:n}\right)p\left(z_{n}|y_{1:n}\right)}{p(y_{n+1:N}|y_{1:n})}\\ =\; & \;p\left(z_{n}|y_{1:n}\right)\int \frac{p\left(z_{n+1}|z_{n}\right)p\left(y_{n+1:N}|z_{n+1},y_{1:n}\right)}{p(y_{n+1:N}|y_{1:n})}\text{d}z_{n+1}\\ =\; & \;p\left(z_{n}|y_{1:n}\right)\int p\left(z_{n+1}|z_{n}\right)\frac{p(z_{n+1}|y_{1:N})}{p(z_{n+1}|y_{1:n})}\text{d}z_{n+1}\\ =\; & \;p\left(z_{n}|y_{1:n}\right)\int p\left(z_{n+1}|z_{n}\right)\frac{p(z_{n+1}|y_{1:N})}{\int p\left(z_{n+1}|z_{n}\right)p\left(z_{n}|y_{1:n}\right)\text{d}z_{n}}\text{d}z_{n+1}. \end{array}$$ (42)

Approximating the integrals using *I* particles, the smoothing weight of particle *i* is

$$\begin{array}{cc} {\bar{w^{*}}}_{n,i} & \approx {\bar{w}}_{n,i}\sum_{j}\frac{p\left(z_{n+1,j}|z_{n,i}\right){\bar{w^{*}}}_{n+1,j}}{\sum_{l}p\left(z_{n+1,j}|z_{n,l}\right){\bar{w}}_{n,l}}, \end{array}$$ (43)

where ${\bar{w}}_{n,i}$ is the normalized filtering weight at time *n* for particle *i*, which is calculated using the bootstrap filter and auxiliary particle filter algorithms introduced in the main text. By a backward sweep, the smoothing weights ${\bar{w^{*}}}_{n,i}$ for *n* = *N*, *N* − 1, … can be recursively computed using the forward filtering weights and the transition probabilities *p*(*z*_*n*+1_|*z*_*n*_) following the state propagation given in (10).

For the semi-online smoothing *p*(*z*_*n*−Δ*n*_|*y*_1:*n*_), at time *n* we proceed the forward filtering to compute the filtering weights, and then run Δ*n* steps backward using (43) to obtain the smoothing weights. The approximation for semi-online smoothing distribution is

$$\begin{array}{c} \hat{p}\left(z_{n-\Delta n}|y_{1:n}\right)=\sum_{i=1}^{I}1_{z_{n-\Delta n}=z_{n-\Delta n,i}}{\bar{w^{*}}}_{n-\Delta n,i} \end{array}$$ (44)

and the posterior mean of the stimulus is estimated as

$$\begin{array}{c} {\hat{S}}_{n-\Delta n}=\sum_{i=1}^{I}s_{n-\Delta n,i}{\bar{w^{*}}}_{n-\Delta n,i}. \end{array}$$ (45)

## References

[1] Dayan P, Abbott LF. Theoretical neuroscience: computational and mathematical modeling of neural systems Computational neuroscience. Cambridge (Mass.), London: MIT Press; 2001 Available from: http://opac.inria.fr/record=b1100424.

[2] Bundesen C, Habekost T, Kyllingsbæk S. A neural theory of visual attention: bridging cognition and neurophysiology. Psychological Review. 2005;112(2):291 10.1037/0033-295X.112.2.291 15783288

[3] Li K, Kozyrev V, Kyllingsbæk S, Treue S, Ditlevsen S, Bundesen C. Neurons in primate visual cortex alternate between responses to multiple stimuli in their receptive field. Frontiers in Computational Neuroscience. 2016;10:141 10.3389/fncom.2016.00141 28082892 PMC5187355

[4] Nobre K, Kastner S. The Oxford handbook of attention. Oxford University Press; 2013.

[5] Lebedev MA, Nicolelis MA. Brain–machine interfaces: past, present and future. TRENDS in Neurosciences. 2006;29(9):536–546. 10.1016/j.tins.2006.07.004 16859758

[6] Waldert S, Pistohl T, Braun C, Ball T, Aertsen A, Mehring C. A review on directional information in neural signals for brain-machine interfaces. Journal of Physiology-Paris. 2009;103(3):244–254. 10.1016/j.jphysparis.2009.08.007 19665554

[7] Georgopoulos AP, Schwartz AB, Kettner RE. Neuronal population coding of movement direction. Science. 1986;233(4771):1416–1419. 10.1126/science.3749885 3749885

[8] Rieke F. Spikes: exploring the neural code. MIT press; 1999.

[9] Warland DK, Reinagel P, Meister M. Decoding visual information from a population of retinal ganglion cells. Journal of Neurophysiology. 1997;78(5):2336–2350. 10.1152/jn.1997.78.5.2336 9356386

[10] Eichhorn J, Tolias A, Zien A, Kuss M, Weston J, Logothetis N, et al Prediction on Spike Data Using Kernel Algorithms In: Thrun S, Saul LK, Schölkopf PB, editors. Advances in Neural Information Processing Systems 16. MIT Press; 2004 p. 1367–1374. Available from: http://papers.nips.cc/paper/2357-prediction-on-spike-data-using-kernel-algorithms.pdf.

[11] Brockmeier AJ, Choi JS, Kriminger EG, Francis JT, Principe JC. Neural decoding with kernel-based metric learning. Neural computation. 2014;26(6):1080–1107. 10.1162/NECO_a_00591 24684447

[12] Koyama S, Eden UT, Brown EN, Kass RE. Bayesian decoding of neural spike trains. Annals of the Institute of Statistical Mathematics. 2010;62(1):37–59. 10.1007/s10463-009-0249-x

[13] Paninski L, Pillow J, Lewi J. Statistical models for neural encoding, decoding, and optimal stimulus design. Progress in brain research. 2007;165:493–507. 10.1016/S0079-6123(06)65031-0 17925266

[14] Pillow JW, Ahmadian Y, Paninski L. Model-based decoding, information estimation, and change-point detection techniques for multineuron spike trains. Neural Computation. 2011;23(1):1–45. 10.1162/NECO_a_00058 20964538

[15] Truccolo W, Eden UT, Fellows MR, Donoghue JP, Brown EN. A point process framework for relating neural spiking activity to spiking history, neural ensemble, and extrinsic covariate effects. Journal of neurophysiology. 2005;93(2):1074–1089. 10.1152/jn.00697.2004 15356183

[16] Kass RE, Eden UT, Brown EN. Analysis of neural data. Springer; 2014.

[17] Wu W, Gao Y, Bienenstock E, Donoghue JP, Black MJ. Bayesian population decoding of motor cortical activity using a Kalman filter. Neural computation. 2006;18(1):80–118. 10.1162/089976606774841585 16354382

[18] Paninski L, Ahmadian Y, Ferreira DG, Koyama S, Rad KR, Vidne M, et al A new look at state-space models for neural data. Journal of computational neuroscience. 2010;29(1-2):107–126. 10.1007/s10827-009-0179-x 19649698 PMC3712521

[19] Kelly R, Lee TS. Decoding V1 neuronal activity using particle filtering with Volterra kernels. In: Advances in neural information processing systems; 2003. p. None.

[20] Brockwell AE, Rojas AL, Kass R. Recursive Bayesian decoding of motor cortical signals by particle filtering. Journal of Neurophysiology. 2004;91(4):1899–1907. 10.1152/jn.00438.2003 15010499

[21] Shoham S, Paninski LM, Fellows MR, Hatsopoulos NG, Donoghue JP, Normann RA. Statistical encoding model for a primary motor cortical brain-machine interface. Biomedical Engineering, IEEE Transactions on. 2005;52(7):1312–1322. 10.1109/TBME.2005.847542 16041995

[22] Li K, Bundesen C, Ditlevsen S. Responses of Leaky Integrate-and-Fire Neurons to a Plurality of Stimuli in Their Receptive Fields. The Journal of Mathematical Neuroscience. 2016;6(1):1 10.1186/s13408-016-0040-2 27215548 PMC4877359

[23] Bundesen C, Habekost T. Principles of visual attention: Linking mind and brain. Oxford Psychology Series, Oxford; 2008.

[24] Townsend JT. Serial vs. parallel processing: Sometimes they look like Tweedledum and Tweedledee but they can (and should) be distinguished. Psychological Science. 1990;1(1):46–54. 10.1111/j.1467-9280.1990.tb00067.x

[25] Fific M, Nosofsky RM, Townsend JT. Information-processing architectures in multidimensional classification: A validation test of the systems factorial technology. Journal of Experimental Psychology: Human Perception and Performance. 2008;34(2):356 10.1037/0096-1523.34.2.356 18377176 PMC2650621

[26] Burkitt AN. A review of the integrate-and-fire neuron model: I. Homogeneous synaptic input. Biological cybernetics. 2006;95(1):1–19. 10.1007/s00422-006-0068-6 16622699

[27] Sacerdote L, Giraudo MT. Stochastic Integrate and Fire Models: A Review on Mathematical Methods and Their Applications In: Stochastic Biomathematical Models with Applications to Neuronal Modeling. vol. 2058 New York: Lecture Notes in Mathematics series (Biosciences subseries), Springer; 2013 p. 99–148.

[28] Kistler W, Gerstner W, Hemmen J. Reduction of the Hodgkin-Huxley equations to a single-variable threshold model. Neural Computation. 1997;9(5):1015–1045. 10.1162/neco.1997.9.5.1015

[29] Gerstner W, Van Hemmen JL, Cowan JD. What matters in neuronal locking? Neural computation. 1996;8(8):1653–1676. 10.1162/neco.1996.8.8.1653 8888612

[30] Fiebelkorn IC, Saalmann YB, Kastner S. Rhythmic sampling within and between objects despite sustained attention at a cued location. Current Biology. 2013;23(24):2553–2558. 10.1016/j.cub.2013.10.063 24316204 PMC3870032

[31] Kantas N, Doucet A, Singh SS, Maciejowski J, Chopin N, et al On particle methods for parameter estimation in state-space models. Statistical science. 2015;30(3):328–351. 10.1214/14-STS511

[32] Box M, Jones MW, Whiteley N. A hidden Markov model for decoding and the analysis of replay in spike trains. Journal of Computational Neuroscience. 2016;41(3):339–366. 10.1007/s10827-016-0621-9 27624733 PMC5097117

[33] Pitt MK, Shephard N. Filtering via simulation: Auxiliary particle filters. Journal of the American statistical association. 1999;94(446):590–599. 10.1080/01621459.1999.10474153

[34] Liu J, West M. Combined parameter and state estimation in simulation-based filtering In: Sequential Monte Carlo methods in practice. Springer; 2001 p. 197–223.

[35] Fearnhead P. Particle filters for mixture models with an unknown number of components. Statistics and Computing. 2004;14(1):11–21. 10.1023/B:STCO.0000009418.04621.cd

[36] Stephens M. Dealing with label switching in mixture models. Journal of the Royal Statistical Society: Series B (Statistical Methodology). 2000;62(4):795–809. 10.1111/1467-9868.00265

[37] Kaufman L, Rousseeuw PJ. Finding groups in data: an introduction to cluster analysis. vol. 344 John Wiley & Sons; 2009.

[38] Hastie T, Tibshirani R, Friedman J, Hastie T, Friedman J, Tibshirani R. The elements of statistical learning. 2nd ed New York: Springer; 2009.

[39] Rios MP, Lopes HF. The extended Liu and West filter: Parameter learning in Markov switching stochastic volatility models In: State-Space Models. Springer; 2013 p. 23–61.

[40] Carvalho C, Johannes MS, Lopes HF, Polson N. Particle learning and smoothing. Statistical Science. 2010;25(1):88–106. 10.1214/10-STS325

[41] Doucet A, Godsill S, Andrieu C. On sequential Monte Carlo sampling methods for Bayesian filtering. Statistics and computing. 2000;10(3):197–208. 10.1023/A:1008935410038

[42] Reynolds JH, Heeger DJ. The normalization model of attention. Neuron. 2009;61(2):168–185. 10.1016/j.neuron.2009.01.002 19186161 PMC2752446

[43] Johansen AM, Doucet A. A note on auxiliary particle filters. Statistics & Probability Letters. 2008;78(12):1498–1504. 10.1016/j.spl.2008.01.032

[44] Douc R, Moulines E, Olsson J. Optimality of the auxiliary particle filter. Probability and Mathematical Statistics. 2009;29(1):1–28.

[45] Whiteley N, Johansen AM. Recent developments in auxiliary particle filtering. Barber, Cemgil, and Chiappa, editors, Inference and Learning in Dynamic Models Cambridge University Press. 2010;38:39–47.

[46] Iolov A, Ditlevsen S, Longtin A. Fokker-Planck and Fortet Equation-Based Parameter Estimation for a Leaky Integrate-and-Fire Model with Sinusoidal and Stochastic Forcing. The Journal of Mathematical Neuroscience. 2014;4(1):4 10.1186/2190-8567-4-4 24742022 PMC4234988

[47] Hurn A, Jeisman J, Lindsay K. ML Estimation of the Parameters of SDEs by Numerical Solution of the Fokker-Planck Equation. In: MODSIM 2005: International Congress on Modelling and Simulation: Advances and Applications for Management and Decision Making; 2005. p. 849–855.

